# Proteomic analysis of trochophore and veliger larvae development in the small abalone *Haliotis diversicolor*

**DOI:** 10.1186/s12864-017-4203-7

**Published:** 2017-10-23

**Authors:** Guilan Di, Xianghui Kong, Xiulian Miao, Yifang Zhang, Miaoqin Huang, Yuting Gu, Weiwei You, Jianxin Zhang, Caihuan Ke

**Affiliations:** 1 College of Fisheries, Henan Normal University, Xinxiang, 453007 China; 2 State Key Laboratory of Marine Environmental Science, Fujian Collaborative Innovation Center for Exploitation and Utilization of Marine Biological Resources, Xiamen University, Xiamen, Fujian Province 361005 People’s Republic of China; 3 College of Life Sciences, Liaocheng University, Liaocheng, 252059 China

**Keywords:** *Haliotis Diversicolor*, Embryonic development, 2-de, Label-free

## Abstract

**Background:**

*Haliotis diversicolor* is commercially important species. The trochophore and veliger are distinct larval stages in gastropod development. Their development involves complex morphological and physiological changes. We studied protein changes during the embryonic development of *H. diversicolor* using two dimensional electrophoresis (2-DE) and label-free methods, tandem mass spectrometry (MS/ MS), and Mascot for protein identification.

**Results:**

A total of 150 2-DE gel spots were identified. Protein spots showed upregulation of 15 proteins and downregulation of 28 proteins as *H. diversicolor* developed from trochophore to veliger larvae. Trochophore and veliger larvae were compared using a label-free quantitative proteomic approach. A total of 526 proteins were identified from both samples, and 104 proteins were differentially expressed (> 1.5 fold). Compared with trochophore larvae, veliger larvae had 55 proteins upregulated and 49 proteins downregulated. These differentially expressed proteins were involved in shell formation, energy metabolism, cellular and stress response processes, protein synthesis and folding, cell cycle, and cell fate determination. Compared with the 5 protein (fructose-bisphosphate aldolase, 14–3-3ε, profilin, actin-depolymerizing factor (ADF)/cofilin) and calreticulin) expression patterns, the mRNA expression exhibited similar patterns except gene of fructose-bisphosphate aldolase.

**Conclusion:**

Our results provide insight into novel aspects of protein function in shell formation, torsion, and nervous system development, and muscle system differentiation in *H. diversicolor* larvae. “Quality control” proteins were identified to be involved in abalone larval development.

**Electronic supplementary material:**

The online version of this article (10.1186/s12864-017-4203-7) contains supplementary material, which is available to authorized users.

## Background

Some of gastropod larval development includes a pelagic phase (trochophore and veliger) and a benthonic phase. The trochophore and veliger are morphologically and behaviorally distinct developmental stages. Complex morphological and physiological processes occur during the transition between these stages. Morphological differences in the development of gastropod larvae have been reported [1–3]. Animal embryology research contributes to the development of the aquaculture industry and environmental pollution monitoring [4]. The embryonic development of gastropods includes several unique features including shell formation and head–foot differentiation [5]. Mollusk development involves shell formation or shell reduction, during which changes in body shape, deposition of minerals, and pigment deposition in a protein matrix occur [6]. The molecular mechanisms underlying these changes are unclear.

Abalones are marine gastropods distributed worldwide along coastal waters in tropical and temperate areas [7]. The small abalone *Haliotis diversicolor* (Mollusca, Gastropoda, Archaeogastropoda) is a commercially important species cultured along coastal waters [8]. In 2010, 50,000 tons of abalone were harvested in China [9].

**Fig. 1:**
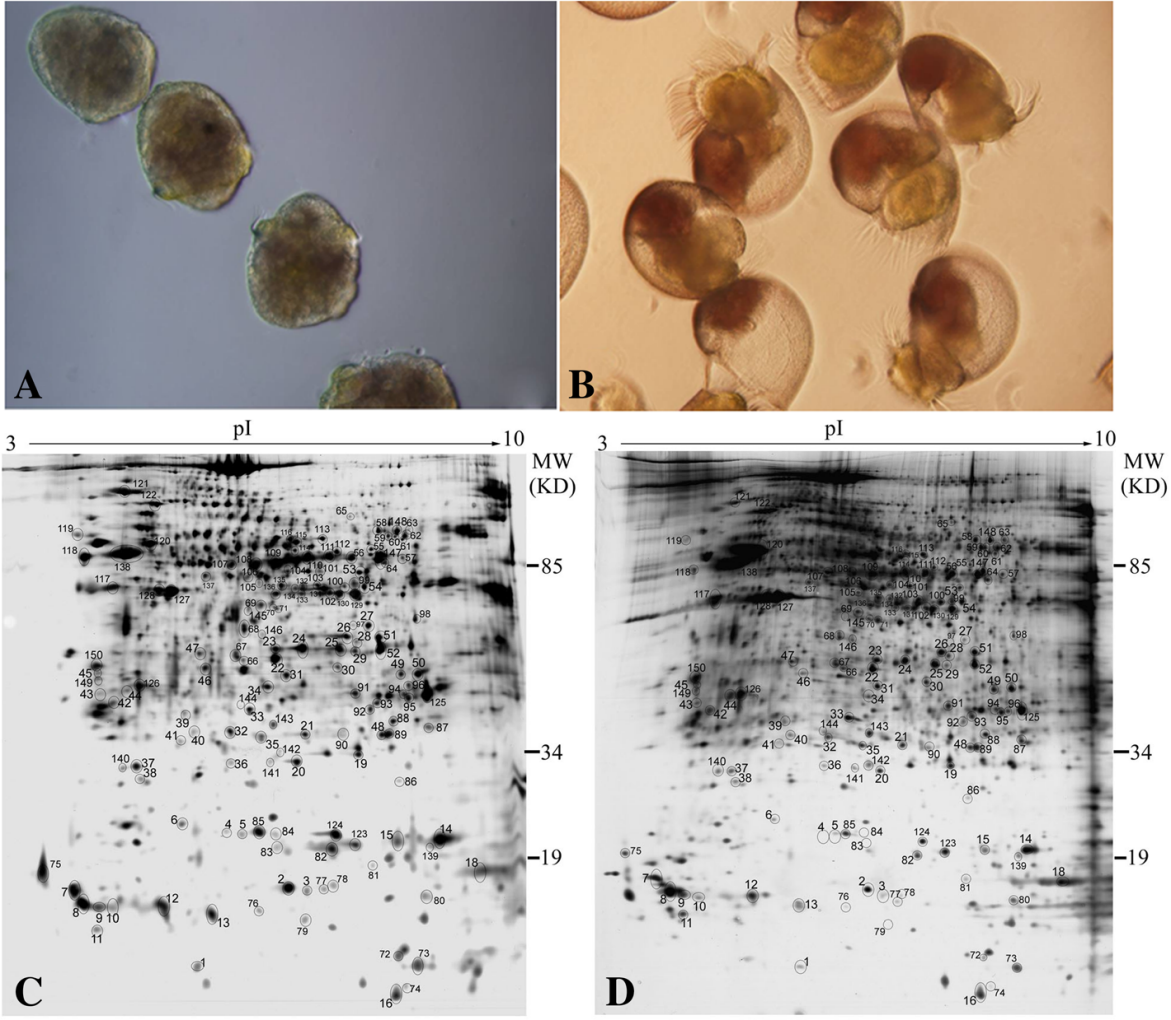
Embryonic development of Haliotis diversicolor and 2-D gel images of silver-stained proteins (120 μg). **a** a trochophore larvae stage, and (**b**) veliger larvae. Larvae hatch as trochophores at about 19 h post-fertilization. The trochophore larvae transform into swimming veliger larvae (at 30 h). Images of 2-DE from (**c**) the trochophore larval proteins; (**d**) the veliger larval stage proteins. The spot-numbering scheme is based on the description in Additional file 2: Table S2

Jackson et al. [1] observed significant differences in developmental events of the abalone *Haliotis asinine.* These included hatching from the vitelline envelope, variation in larval shell development, and metamorphosis-inducing cues in older larvae. While many proteins are involved in abalone shell formation, their presence and roles in early developmental stages of larval shell formation are not well understood [2, 3]. Larval torsion of the shell is also important in the development of gastropods. The abalone trochophore and veliger larvae possess shell at the different stages of growth. Larvae hatch as trochophores, at approximately 19 h post-fertilization (Fig. 1a), and the trochophore is the initial shell stage. The trochophore then undergoes a series of morphological changes, including velum acquisition, at which point it becomes a swimming larva in the pre-veliger phase (at 30 h). The trochophore then transforms into a swimming veliger larva, and the late calcified protoconch forms (Fig. 1b). The veliger stage is characterized by differentiation of the larval retractor muscle, foot mass, mantle, and the onset of shell mineralization [10, 11].

Sequencing techniques, genomic data, and transcriptomic analysis have been used to study larval development, the transcriptome of the early life history stages of the California Sea Hare *Aplysia californica* has been reported [12]. However, current genomic data are insufficient to reveal the molecular mechanisms underlying the complex cellular processes of embryonic development [13]. The process by which shell matrix proteins are secreted and organized into larval shell architecture is largely unknown. Proteins determine phenotypes, which can be considered snapshots of genome expression [14]. Phenotypes are more complex than mere genomic expression, due to lack of a direct correlation between gene expression intensity and protein abundance, proteomic studies are powerful tools for the discovery of new proteins involved in developmental processes. Proteomic profiling has been applied to embryo studies of several invertebrates, including fruit flies (*Drosophila melanogaster*) [15], brine shrimps (*Artemia franciscana*) [16], honey bees (*Apis mellifera*) [17], fouling barnacles *(Balanus amphitrite*) [18, 19], polychaetes (*Pseudopolydora vexillosa*) [20], ascidians (*Ciona intestinalis*) [21], golden apple snail (*Pomacea canaliculata*) [22], and Pacific oyster (*Crassostrea gigas*) [13].

Two-dimensional electrophoresis (2-DE) permits quantitative examination within a single gel electrophoresis experiment and it is a powerful tool for total protein separation. Matrix-assisted laser desorption/ionization (MALDI) mass spectrometry is a promising technique [23]. Label-free-based proteomics is broadly applied to biomarker discovery and proteomic profiling [24]. This technique is rapid, clean and simple. It can directly and precisely quantify protein expression without using labeling. Proteomic studies of abalone embryos have not been previous reported. To thoroughly evaluate larval proteome changes, we investigated the differential protein abundances during the different development stages of *H. diversicolor* using a label-free, coupled 2-DE proteomic approach. The results provide insights into the different protein groupings during shell formation and the differentiation of the larval retractor muscle, foot mass, and mantle.

## Methods

### Animals

Adult *H. diversicolor* were obtained from Dongshan Haitian Aquaculture in Fujian Province, China. Abalones were fed on red seaweed *Gracilaria* sp., and the water temperature was maintained at 26–28 °C. Fertilized eggs were incubated for 19 and 38 h to allow development into trochophores and veligers, respectively. At each stage, almost all individuals were in the same phase of development as confirmed by microscopic examination. Fresh, living larvae, approximately 4000–5000 per stage, were filtered on a 40 μm filter and then collected in 1.5 mL tubes for further analysis. The trochophores (C1) (0.192–0.216 × 0.155–0.173 mm) and veligers (C2) (0.215–0.235 × 0.170–0.185 mm) were centrifuged for 3 min at 13,000×*g*. The larval material (15 μL of pellets) was lysed in a 1.5 mL tube by using 1 mL of TRIzol and then stored at −80 °C. The trochophore (or veliger) larval material was divided into three independent samples, and three technical replicates were performed for each sample to ensure reproducibility.

### Extraction of abalone proteins

Protein extractions were performed using TRIzol-mediated sample preparation as described previously [25]. The protein pellet was air-dried and resuspended in an isoelectric focusing (IEF) re-dissolving buffer (7 M urea, 2 M thiourea, 4% [*w*/*v*] CHAPS, and 40 mM Tris, pH 8.5). Protein concentration was measured using a Protein 2-D Quant kit (GE Healthcare). Samples were stored at −70 °C prior to experiments.

### 2-de

A 2-DE was conducted as described previously [26]. Total protein (120 μg) was used for each test. The first dimension (IEF) of gel electrophoresis was conducted on 18 cm immobilized pH gradient (IPG) strips using a nonlinear pH gradient (pH 3–10, Amersham Pharmacia Biotech) with a horizontal electrophoresis apparatus (Bio-Rad). The sample, included in the rehydration solution was loaded on the IPG (Immobilized pH Gradient) strip holder. IPG dry strips were rehydrated with a rehydration buffer (8 M urea, 2% (*w*/*v*) CHAPS, 20 mM DTT, 0.5% (*v*/v) IPG buffer 3–10, and 0.01% (w/v) bromophenol blue, pH 7.4) at 50 V. IEF was then initiated at 100 V for 2 h, 200 V for 2 h, 500 V for 1 h, 1000 V for 2 h, 4000 V for 2 h, and finally 8000 V until 50,000 Vh. Before the second dimension, the IPG strips were soaked for 17 min in equilibration solution (6 M urea, 50 mM Tris-HCI buffer, pH 8.8, 30% v/v glycerol, 2% w/v SDS, and a trace of bromophenol blue) containing 1% w/v DTT. Incubation was continued with equilibration solution containing 2.5% (w/v) iodoacetamide for an additional 17 min. The second dimension of gel electrophoresis was conducted on 12.5% polyacrylamide gels (20 cm × 20 cm × 1.5 mm) by using the protean Xi Cell (Bio-Rad). Equilibrated strips were placed onto gels to perform sodium dodecyl sulfate–polyacrylamide gel electrophoresis at 16 °C. The separation was conducted at 12.5 mA/gel for 30 min and then 25 mA/gel for approximately 5.5 h until the dye front reached the gel bottom. The protein spots were visualized with silver nitrate.

### Image acquisition and analysis

For each development period, three independent samples were used in 2-DE analysis, and three technical replicates were conducted for each sample. The 2-DE gels were scanned to create TIFF files using an image scanner (Amersham Biosciences, UTA-1100). Differences in spot intensity were analyzed with the PDQuest 8.0 software package (Bio-Rad) according to manufacturer instructions. All gels were matched with a selected reference gel. The following corrections were performed so that only true spots were identified. The background was subtracted and edited to correct possible errors by removing inaccurate spots. Spot intensities of stained gel images were quantified according to total spot density and normalized to total gel density using PDQuest. Spot intensity levels were normalized by expressing the intensity of each protein spot as a proportion of the total protein intensity in a gel (relative volume, % vol). Only well-resolved spots were used; spots in overlapping areas, streaked areas, or near the edges were discarded.

### Statistical analysis

Differences between the two larvae stage groups were compared using SPSS version 17.0 software. We tested the null hypothesis that the difference of spot amounts had a mean of zero. Spot analysis was performed using both qualitative and quantitative modes. Spot intensity analysis was performed using the two-sided Student’s t-test. The *p*-value for each individual spot was adjusted to account for multiple testing using the false discovery rate (FDR) method [27]. A cutoff of 0.11 was used for the FDR to assess significance of results. This cutoff rate indicates that 11% of the spots with significant values are expected to be false positives [28]. An error probability of q < 0.05 was considered to be statistically significant. For quantitative analysis, only protein spots showing at least a 1.5-fold change among the three groups were compared in the PDQuest software and considered up-regulated or down-regulated in order to compensate for the technical variability between replicates. Protein spots detected quantitatively were compared between ‘two groups’. For qualitative analysis, spots with at least 10-fold changes were considered absent/present [29].

### Mass spectrometric protein identification

Protein identification was conducted as described previously [25]. Briefly, protein spots were excised from the 2-DE gel and digested with trypsin. The excised spots were washed for 10 min with water, then the spots were destained using 15 mM potassium ferricyanide, 50 mM sodium thiosulfate, and washed using 200 L Milli-Q H_2_O (5 min × 3). Spots were then shaken in 200 μl 200 mM NH_4_HCO_3_ solution for 20 min, dehydrated using 200 μl acetonitrile for 10 min, and then the acetonitrile (ACN) was removed. The gel pieces (protein spots) were dried. Proteins were reduced by treatment with 10 mM DTT for 30 min at 56 °C, and alkylated with 55 mM iodoacetamide. Trypsin digestion was performed by addition of 4 μl of trypsin (20 ng/L) in 20 mM NH_4_HCO_3_ solution to each spot followed by incubation overnight at 37 °C. The extract (0.5 μL) was analyzed using MALDI/time-of-flight/time-of-flight (MALDI-TOF/TOF) with a 5800 Proteomics Analyzer (Applied Biosystems); the MALDI conditions were identical to those given in the literature [26]. The extracts were pooled, dried, and resuspended in the extracts using 5 μL of 50% (*v*/v) ACN and 0.1% (v/v) trifluoroacetic acid (TFA). These liquids (0.8 μL) were mixed with 0.3 μL of matrix solution (2 μg/μL R-cyano-4-hydroxycinnamic acid) in 50% (v/v) ACN and 0.1% (v/v) TFA. Proteins were identified from the peptide mass fingerprints obtained by MALDI-TOF/TOF. From the results of tandem mass spectrometry (MS/MS), up to 2000 laser shots were acquired, and peptides were fragmented with collision-induced decomposition at an energy of 1 kV. The 20 most intense precursors per spot were selected with a minimum signal-to-noise (S/N) ratio of 50, and the peak detection criteria used a minimum S/N of 10.

### Mass spectrometric data analysis

MS/MS peaks were retrieved using an in-house Mascot Distiller. Data files were screened against the protein database for identification with Mascot. A protein identification was accepted if it contained at least two identified peptides both having a minimal cutoff Mascot score of 24. Peptides identified with a probability of 95% correct matches were considered as “significant sequences.” Combined MS and MS/MS searches were conducted against the nrNCBI database. The search parameters were as follows: the enzyme was trypsin; the allowance was one missed cleavage site; the fixed modification was carbamidomethyl (cysteine); the variable modification was Met oxidation; monoisotopic mass values were obtained; protein mass was unrestricted; the peptide mass tolerance was ±100 ppm; the fragment mass tolerance was ±0.5 Da. Similarities were considered significant when the total ion C.I. % was ≥95, and the E value was below e^−20^. The decoy search was performed to estimate the false discovery rate (FDR).

The database used in this study was UniProtKB. The Gene Ontology (GO) database included vocabularies, contributed annotations, and provided full access to this information in several formats. GO and prediction subcellular localization were analyzed using http://www.uniprot.org/uniprot/. Predicted interactions of the identified differentially expressed proteins were made using the http://string.embl.de/ website.

### Filter-aided sample preparation protein digestion for label-free proteomic analysis

The protein pellet was dissolved in a solution of urea (6 M) and thiourea (2 M). Proteins were reduced with dithiotreitol (1 mM) for 30 min, followed by alkylation with iodoacetamide (55 mM) for 30 min in darkness. Incubation with trypsin (1 g/50 g protein) (Promega, Madison, WI) was conducted for 12 h at room temperature. The digestion was stopped by addition of 5% formic acid. The peptide mixture was desalted using reversed phase C18 Stage Tips (Thermo Fisher Scientific), then washed three times with 50% ACN, 0.1% TFA; the peptide elutions were combined. Peptide concentration was determined using a NanoDrop spectrophotometer [30]. Peptides were frozen at −70 °C.

### Label-free liquid chromatography (LC)-MS/MS Q Exactive quantification

The reversed-phase LC column was 5 μm, with 200 Å pore size C18 resin (Hypersil Gold C18, Thermo Fisher Scientific, Bremen, Germany) in a 75 μm i.d. × 10 cm. After injecting the sample, the column was washed for 5.5 min with 90% mobile phase A (0.1% formic acid in water) and 10% mobile phase B (0.1% formic acid in ACN). The peptides were eluted using a linear gradient of 10%–50% mobile phase B for 24 min and then 50%–80% mobile phase B for 6 min at a flow rate of 300 nL/min, a column temperature of 30 °C, and an injection volume of 10 μL. At least a 5 μg sample of each digest was used, and each sample was analyzed three times.

Data-dependent label-free analysis was performed with three replicate injections of each sample using a Q Exactive mass spectrometer (Thermo Scientific) at a flow rate of 300 nL/min. The data-dependent program used for data acquisition consisted of a 70,000 resolution full-scan MS scan (AGC set to 10^6^ ions with a maximum fill time of 200 ms), and the 10 most abundant peaks were selected for MS/MS using a 17,500 resolution scan (AGC set to 1 × 10^4^ ions with a maximum fill time of 200 ms) with an ion selection window of 1.6 mass-to-charge (m/z) ratio and a normalized collision energy of 30 eV. The under-fill ratio, which assigns the minimum percentage of the target value likely to be reached at maximum-fill time, was regarded as 0.1%. The instrument was run with the peptide recognition mode enabled [31]. The program used a 40 s dynamic exclusion window to avoid repeated selection of peptides for MS/MS. Survey scans were acquired at a resolution of 70,000 at 200 m/z, and resolution for HCD spectra was set to 17,500 at 200 m/z.

### Data analysis for label-free proteomic analysis

Raw files were processed to peak lists. Raw spectra were converted to Mascot-generated files using Proteome Discoverer software (Thermo Scientific) and analyzed using SIEVE software, which quantified all detected peaks. The q-value was used to estimate false positive results. All data were obtained based on 99% confidence for protein identification by FDR ≤1%. Statistical analysis was performed using a one-way ANOVA. *P*-values ≤ 0.05 obtained by Tukey’s test were considered significant. Protein abundances that changed less than 1.5-fold were discarded [31].

We compared differentially expressed proteins in C1 and C2 with a correlation test using scatterplot. A heat map colour column was constructed based on the values of the differential proteins in two groups using HemI 1.0 statistical software.

### Experimental validation using RT-qPCR

The differentially expressed protein genes were validated using RT-qPCR amplification to confirm the proteomic results. Quantitative real-time PCR was conducted using an ABI Quantstudio 6 Flex system with SYBR ® Premix Ex Taq™ (TaKaRa, Japan) according to manufacturer instructions. Primer sequences were designed based on each identified gene sequence using Primer Premier 6 software (Premier Biosoft, USA) (Additional file 1: Table S1). PCR amplification experiments were conducted in triplicate under the following conditions: 95 °C for 30 s, then 40 cycles of 95 °C for 5 s, 55 °C for 30 s, and 72 °C for 30 s. The results were normalized using 18sRNA and Y-box protein 1(GenBank accession no.JN997407.1) for each sample and the 2^-△△CT^ method. The expression levels of each gene in the two developmental stages were compared using a two-sided Student’s t-test, and differences were considered statistically significant at *p* < 0.05.

## Results

### Identification of 2-DE spots

Different proteins were separated by 2-DE, as shown in Fig. 1c-d. A total of 150 gel spots were identified. The identification results from MS/MS are summarized in Additional file 2: Table S2. The protein spots identified by MS/MS in the abalone larvae with normalized % spot volumes calculated from the protein differential abundances, functions, and processes are summarized in Additional file 3: Table S3.

**Fig. 2:**
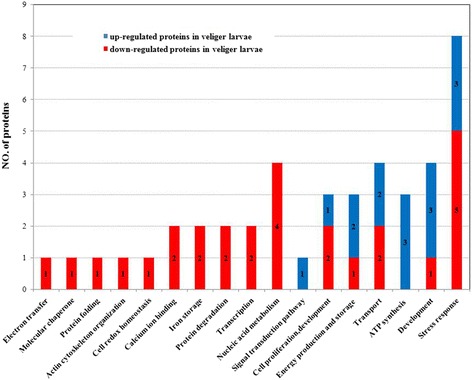
The GO functional categories of the up-regulated or down-regulated differential abundances proteins based on 2-DE. Color codes are number of proteins identified by different analysis

Among the differential abundance proteins identified, proteins from 15 GO functional categories were upregulated and proteins from 28 GO functional categories were downregulated between the protein samples from the trochophores and veligers (Fig. 2). The most of the upregulated proteins were involved in development (3 proteins including the 14–3-3 epsilon protein, 3-monooxygenase/tryptophan5-monooxygenase activation protein, and the metal-dependent hydrolase [32–35]), stress response (3 proteins including the receptor of activated kinase C, glutathione-S-transferase isoform, and manganese-superoxide dismutase), ATP synthesis (3 proteins including GK21455, vacuolar proton-ATPase E-subunit, and ATP synthase F1), carbohydrate metabolism (2 proteins including SJCHGC09380 protein and citrate synthase 1), and transport (2 proteins including charged multivesicular body protein 4c and enoyl-CoA hydratase). Most downregulated proteins were involved in stress response (5 proteins including thioredoxin peroxidase 1, thiol peroxiredoxin and Cu, Zn-superoxide dismutase), nucleic acid metabolism (4 proteins including dUTPase and inosine 5′-phosphate dehydrogenase 1), cellular proliferation, development (2 proteins including actin depolymerisation factor/cofilin), calcium ion binding (2 proteins including calreticulin and calmodulin 2), transport (2 proteins including outer membrane protein A precursor and charged multivesicular body protein 4c), iron storage (2 proteins including ferritin and soma ferritin), protein degradation (2 proteins including BRAFLDRAFT_260175 and ubiquitin carboxyl-terminal hydrolase 14), and transcription (2 proteins including similar to pterin-4a-carbinolamine and prevent-host-death family protein).

### Label-free proteomic analysis

**Fig. 3:**
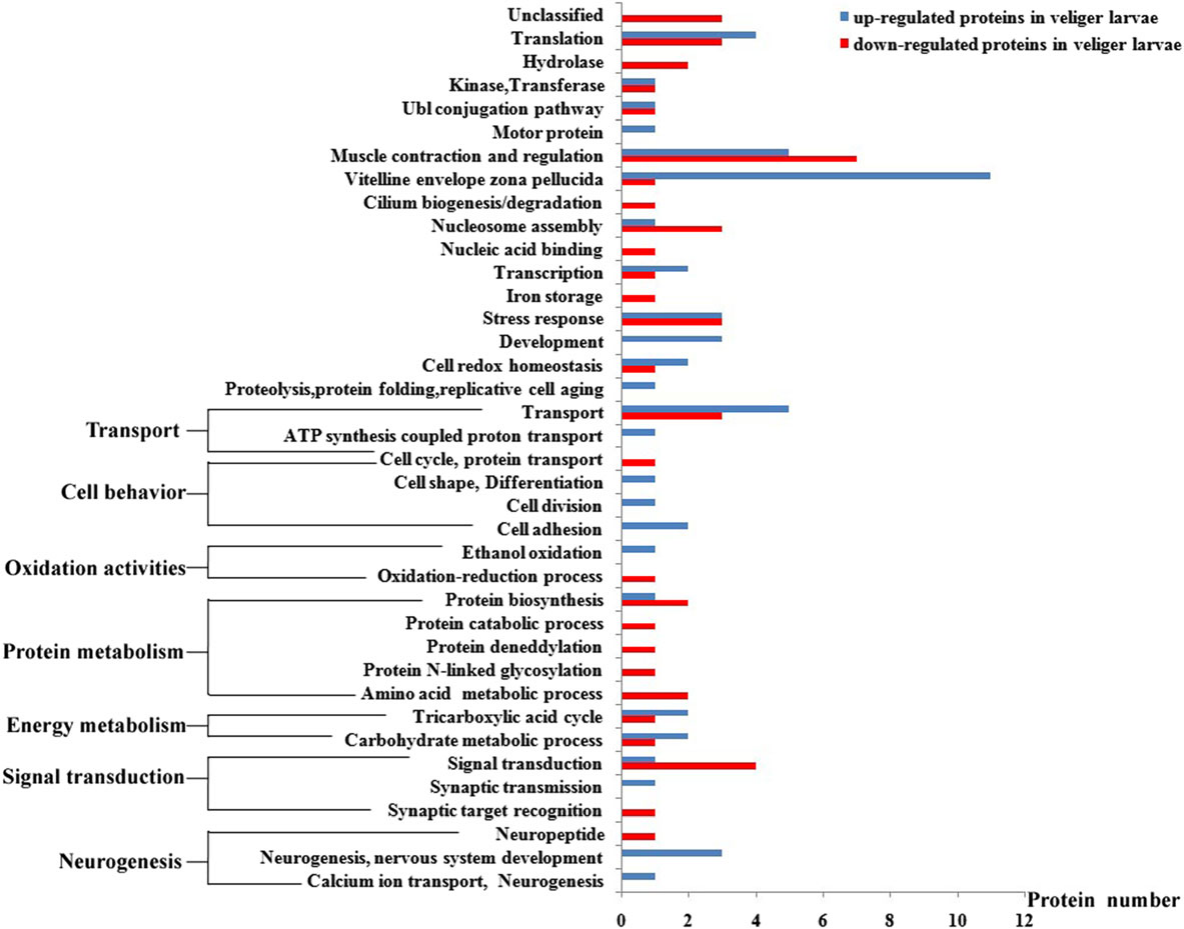
GO analysis for differentially abundant proteins of veliger larvae based on label-free analysis

A total of 526 proteins (FDR < 1%, at least two unique peptides) were identified from both of the samples, and 104 differential abundance proteins (> 1.5-fold) were detected using the label-free comparative proteomic approach. The veligers had 55 upregulated and 49 downregulated proteins compared with the trochophores (Additional file 4: Table S4). The upregulated proteins were involved in vitelline envelope zona pellucida, muscle contraction and regulation, translation, development, neurogenesis, nervous system development, stress response, and cell redox homeostasis (Fig. 3). The downregulated proteins were involved in muscle contraction and regulation, signal transduction, stress response, translation, and transport. As shown in Fig. 3, the protein abundance profiles differed significantly in the trochophore and the veliger larval stages.

**Fig. 4:**
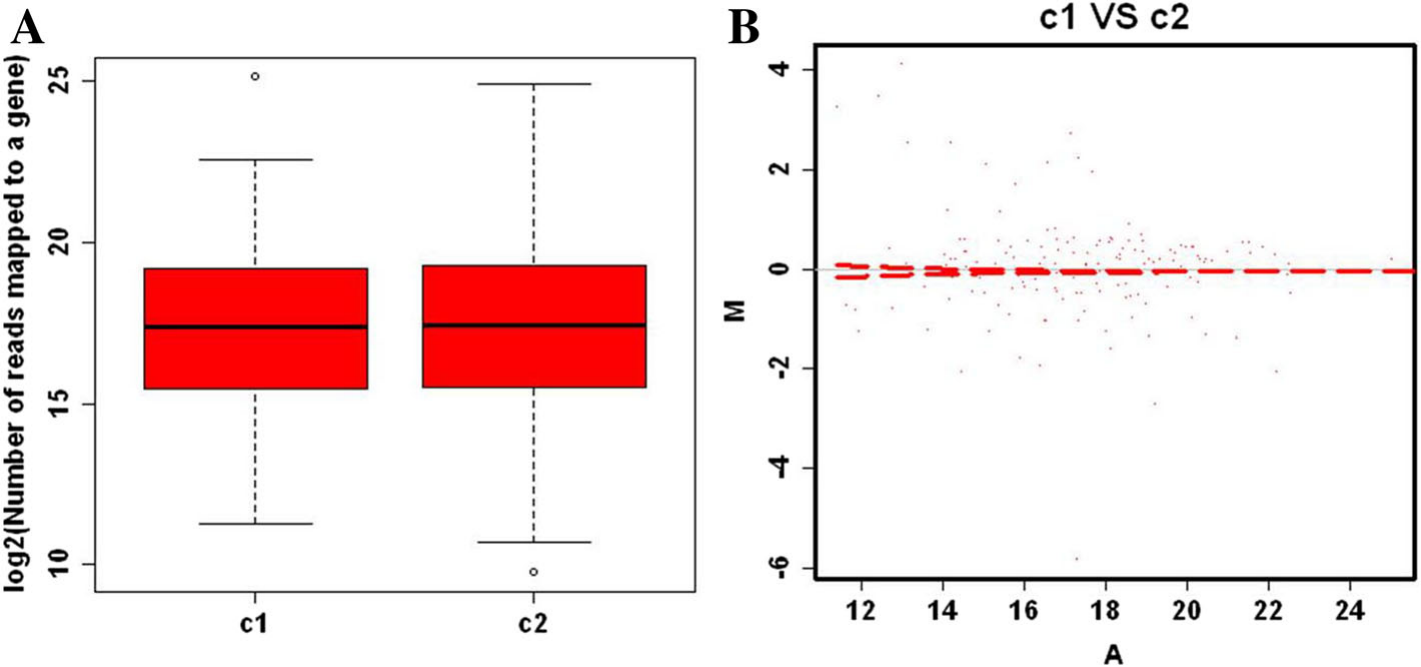
Boxplot showing the location and the spread of differentially expressed genes of differentially abundant proteins. **a** Boxplot of read counts for each gene. **b** Differentially expressed genes on the MA-plot

The boxplot shows information about the location and the spread of the data by means of the median and the interquartile range. To show the location and the spread of differentially expressed genes of different proteins in the two sample groups (trochophore and veliger larvae), we used a boxplot. A boxplot of read counts for each gene in the trochophore larvae (C_1_) and veliger larvae (C_2_) is shown in Fig. 4a. The measurements for C_2_ were more variable than C_1_, but no significant differences were observed. Differentially expressed genes (red points) in the MA-plot (a graphical method for visualizing intensity-dependent ratio of raw microarray data) are shown in Fig. 4b.

**Fig. 5:**
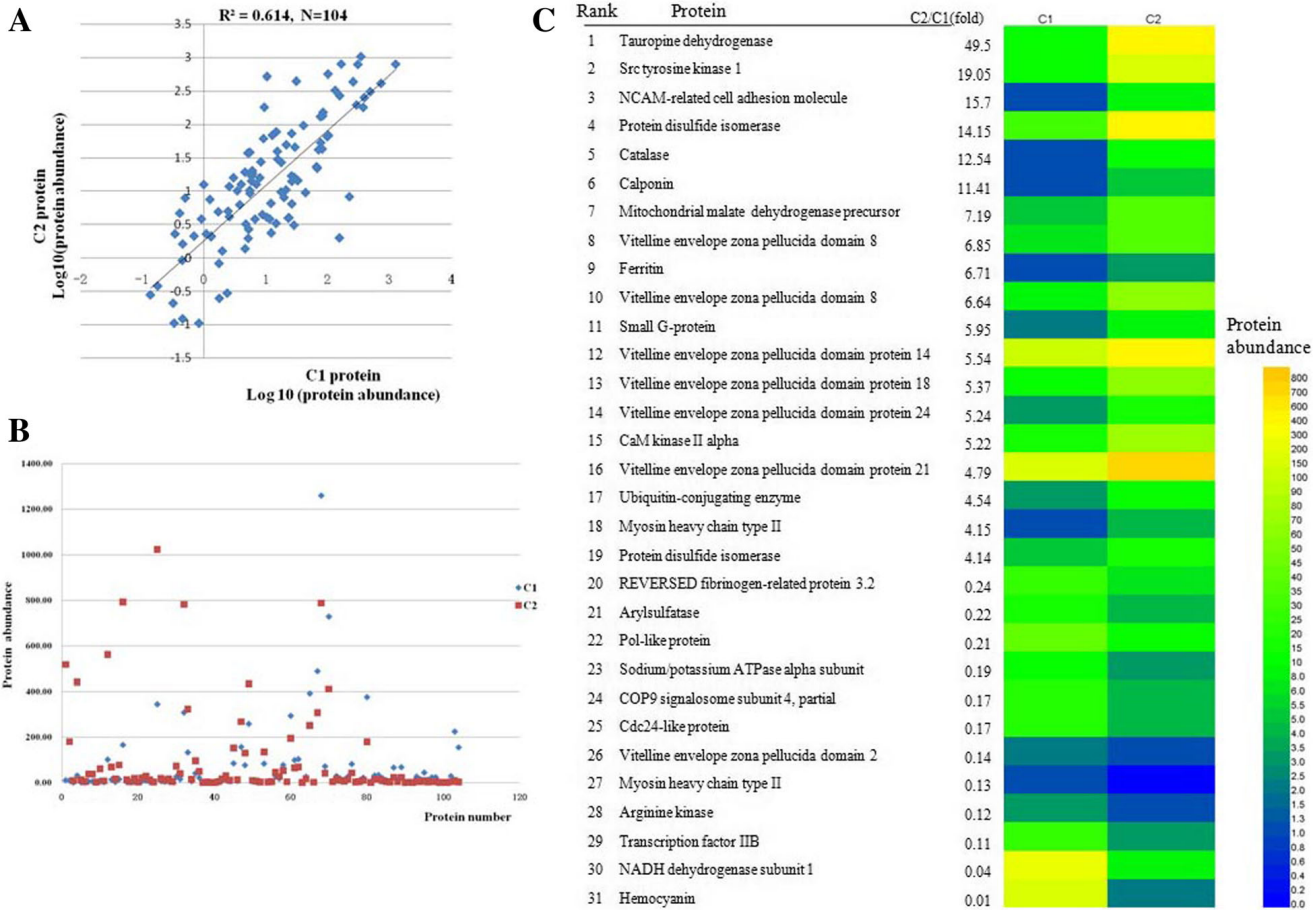
Overlaps in ranks of proteins between C1 and C2. **a** Correlation between C1 and C2 proteins based on concentrations of 104 proteins; (**b**) The abundance of 104 differentially proteins; (**c**) Rank of top 31 abundant proteins

To test whether there is a direct correlation in corresponding proteins, we compared the estimated concentrations of differentially expressed proteins in C_1_ and C_2_ with a correlation test (Fig. 5a). The correlation across the two stages was relatively low. Yet, decent positive correlation was still observed between C_1_ and C_2_, with a correlation score of 0.614. To compare the abundance ranking of differentially proteins between C_1_ and C_2_, the abundance of 104 differentially proteins are showed in Fig. 5b. Among the top 31 most abundant proteins identified (Fig. 5c), 15 differentially expressed proteins increased over 5 fold in C_2_ and nine differentially expressed proteins decreased over 5 fold in C_2_.

### Comparisons of differential abundance proteins

**Fig. 6:**
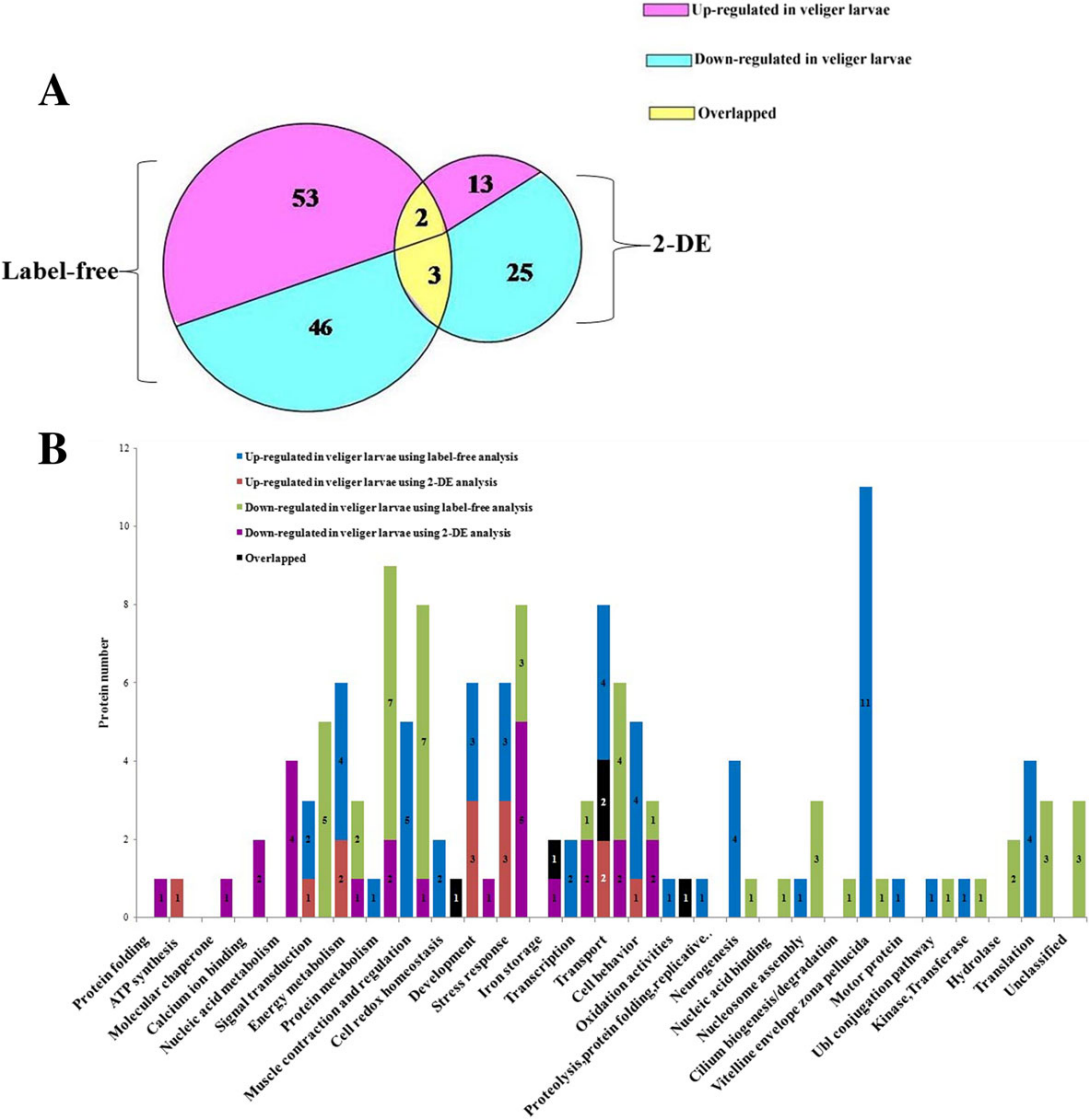
Comparison of the differential proteins identified by 2-DE and label-free LC–MS analysis. **a** Venn diagram showing the distribution of 142 proteins. The large pie chart represents the proteins identified by the label-free LC–MS-based technique, and the small pie chart represents the proteins identified by the 2-DE technique. The numbers indicate the protein numbers in each partition. **b** Qualitative comparison of the protein numbers in veliger larvae. A total of 142 differentially abundant proteins are grouped into 29 categories according to their biological functions. Color codes are the protein numbers identified by differential analysis

There were 142 nonredundant proteins with differential abundances between the trochophore and veliger larvae determined by both 2-DE and label-free LC–MS-based techniques. Of these, 38 and 99 proteins were exclusively identified and 5 proteins overlapped (Fig. 6a). In the veliger, 68 (47.89%) proteins were upregulated and 74 (52.11%) proteins were downregulated. The differentially expressed proteins were mainly related to transport (14), stress response (14), muscle contraction and regulation (13), protein metabolism (10), energy metabolism (9), signal transduction (8), and development (7) (Fig. 6 b).

### Prediction subcellular localization of protein

**Fig. 7:**
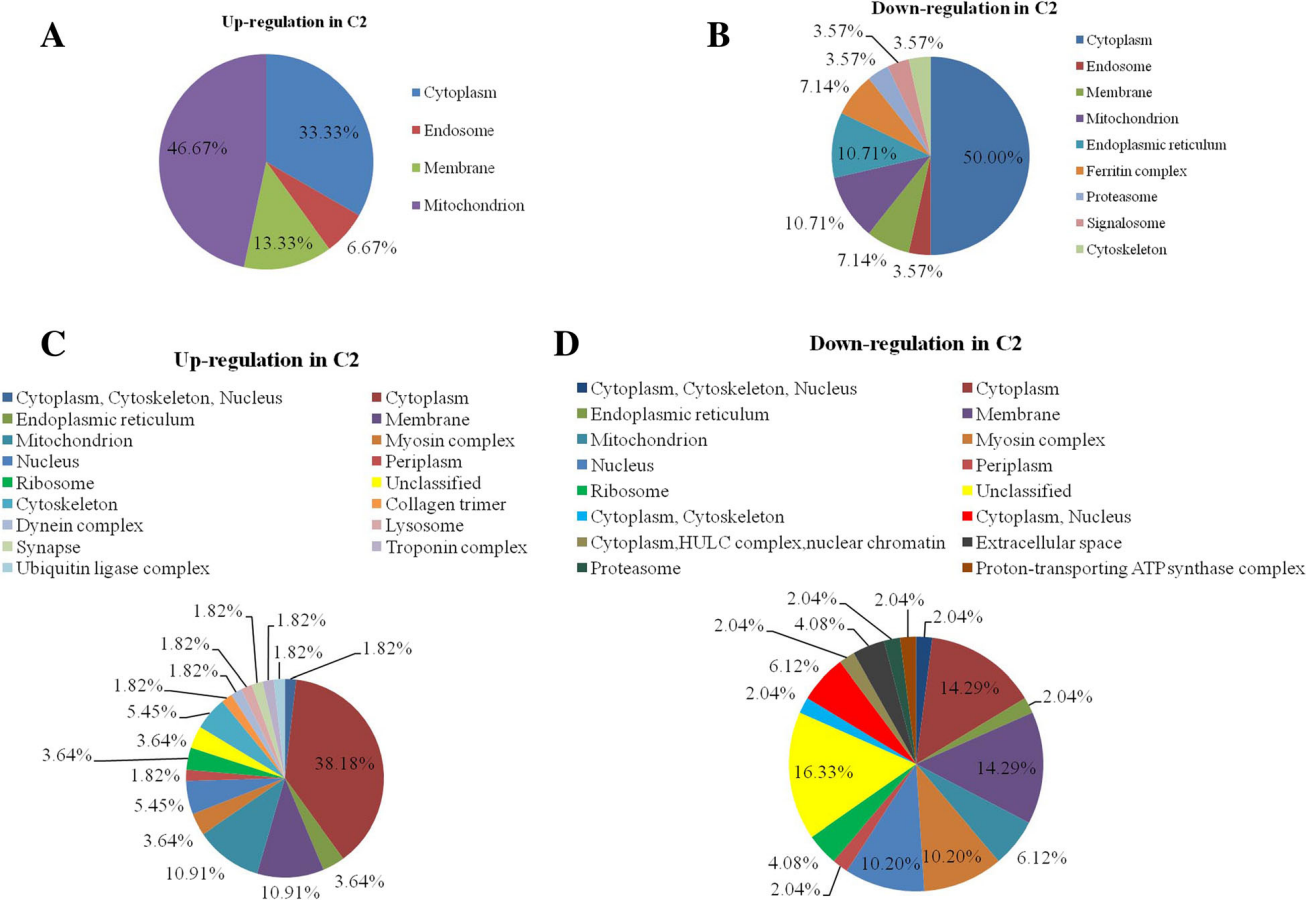
Pie chart showing the distribution of the differentially expressed proteins identified according to subcellular localization. **a** The subcellular locations of upregulated proteins in C2 by the 2-DE technique; (**b**) The subcellular locations of downregulated proteins in C2 by the 2-DE technique; (**c**) The cellular component of upregulated proteins in C2 based on label-free analysis; (**d**) The cellular component of downregulated proteins in C2 based on label-free analysis

The predicted subcellular locations of differentially expressed proteins are given in Fig. 7. The cellular components of 43 differentially expressed proteins in C_2_ (by 2-DE gel analysis) are shown in Fig. 7a-b. The subcellular locations of upregulated proteins in C_2_ were showed in Fig. 7 a, downregulated proteins in C_2_ were showed in Fig. 7 b.

Cellular components of 104 differentially expressed proteins in C_2_ (by label-free analysis) are shown in Fig. 7 c-d. Upregulated cellular components were showed in Fig. 7c, downregulated cellular component were showed in Fig. 7d. A significant portion of the upregulated proteins in C_2_ was in the collagen trimer (1.82%), dynein complex (1.82%), lysosome (1.82%), synapse (1.82%), troponin complex (1.82%), ubiquitin ligase complex (1.82%). A significant portion of the downregulated proteins in C_2_ was in the cytoplasm, HULC complex, nuclear chromatin (2.04%), extracellular space (4.08%), proteasome (2.04%), and proton-transporting ATP synthase complex (2.04).

### Predicted interactions of identified differential proteins from larvae

**Fig. 8:**
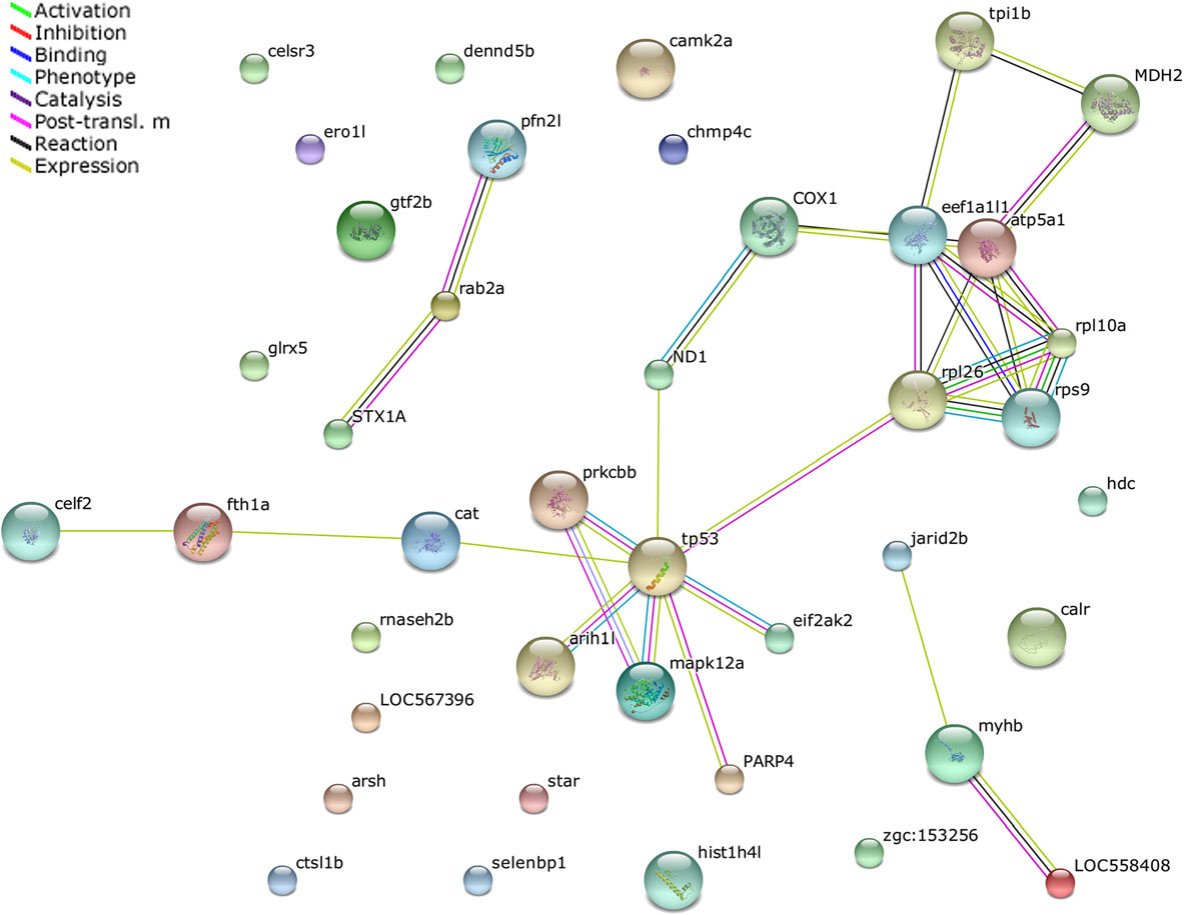
Predicted interactions of identified differentially expressed proteins. Protein abbreviations and corresponding full name are shown: LOC558408 (troponin I); rab2a (RAB2A, member RAS oncogene family); myhb (myosin, heavy chain b); mapk12a (mitogen-activated protein kinase 12a); cat (catalase); fth1a (ferritin, heavy polypeptide 1a); atp5a1 (ATP synthase, H+ transporting, mitochondrial F1 complex, alpha subunit 1, cardiac muscle); prkcbb (protein kinase C, beta b); PARP4 [poly (ADP-ribose) polymerase family, member 4]; tp53 (tumor protein p53); arih1l (ariadne homolog, ubiquitin-conjugating enzyme E2 binding protein, 1 like); rpl26 (ribosomal protein L26); tpi1b (triosephosphate isomerase 1b); MDH2 [malate dehydrogenase 2, NAD (mitochondrial)]; ND1(NADH dehydrogenase 1, mitochondrial); COX1 (cytochrome c oxidase I, mitochondrial)); eif2ak2 (eukaryotic translation initiation factor 2-alpha kinase 2); celf2 (cugbp, Elav-like family member 2); eef1a1l1 (eukaryotic translation elongation factor 1 alpha 1, like 1); pfn2l (profilin 2 like); jarid2b (jumonji, AT rich interactive domain 2b); STX1A [syntaxin 1A (brain)]; rps9 (ribosomal protein S9); rpl10a (ribosomal protein L10a)

**Fig. 9:**
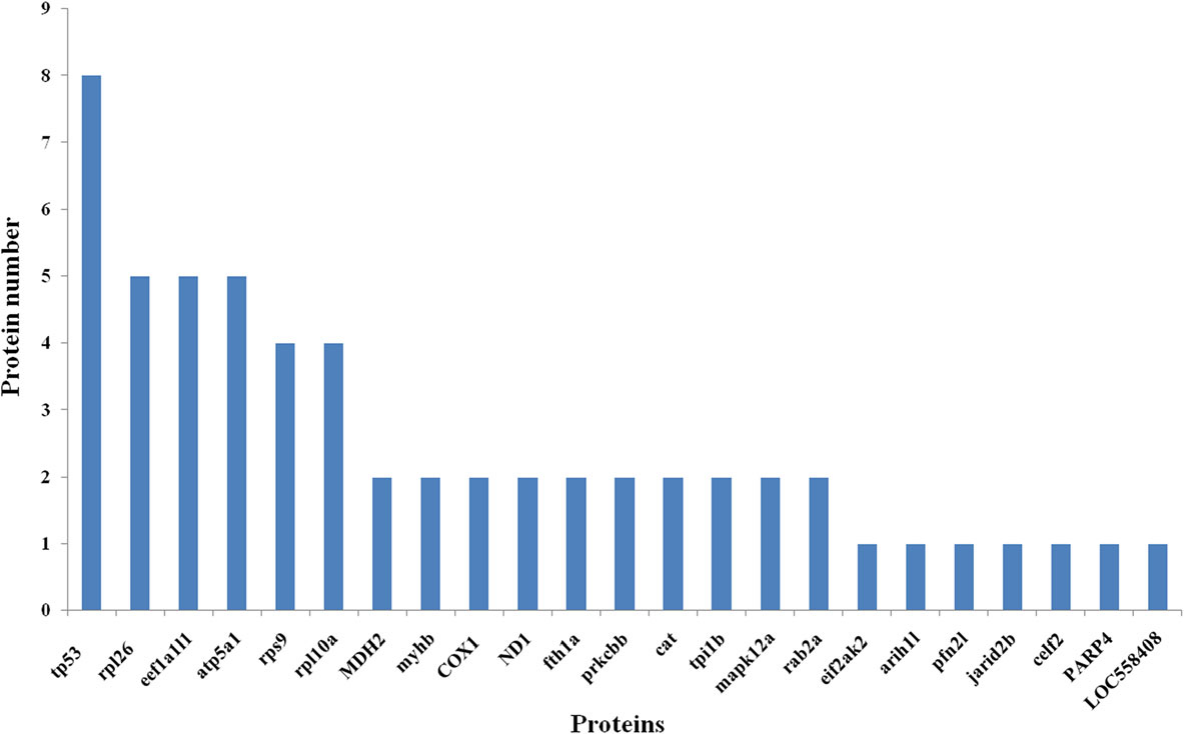
Interactive network of protein co-expression

Predicted interactions are shown in Fig. 8. The number of interactive networks of protein co-expression is shown in Fig. 9. The differentially expressed co-expression proteins mainly included tumor protein p53 (tp53); ribosomal protein L26 (rpl26); eukaryotic translation elongation factor 1 alpha 1, like 1 (eef1a1l1); ATP synthase, H+ transporting, mitochondrial F1 complex, alpha subunit 1, cardiac muscle (atp5a1); ribosomal protein S9 (rps9); and ribosomal protein L10a (rpl10a).

### Comparison of protein expression and mRNA transcription

**Fig. 10:**
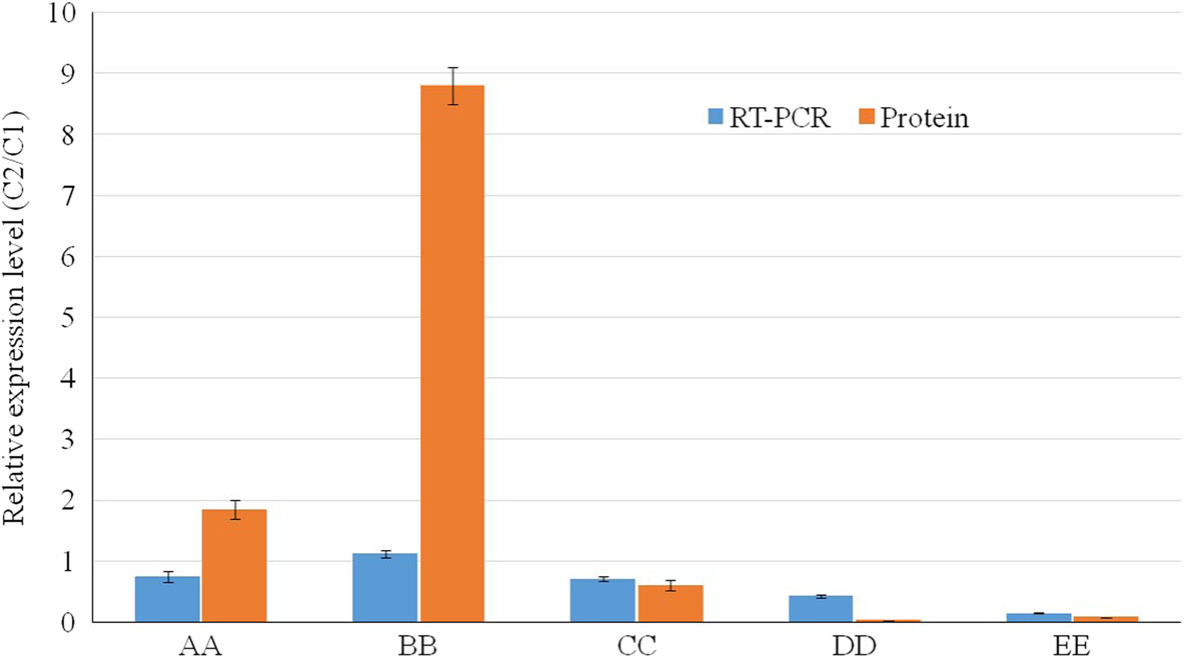
Comparison of mRNA and protein expression levels for five genes in trochophore and veliger larvae. Values in each column represent the relative expression level of the mRNA or protein. Values greater than 1 represent up-regulations in veliger larvae, and values less than one indicate down-regulations. Four of the genes exhibited the same trend, but a discrepancy was observed between the mRNA expression and the protein level of the AA gene. AA = Fructose-bisphosphate aldolase; BB = 14–3-3 ε; CC = Profiling; DD = Actin-depolymerizing factor (ADF)/cofilin; EE = Calreticulin

To investigate the transcriptional levels of identified proteins, five of the ESTs matched by proteins (AA = fructose-bisphosphate aldolase, BB = 14–3-3ε, CC = profilin, DD = actin-depolymerizing factor (ADF) / cofilin) and EE = calreticulin) were chosen for real-time PCR analysis. Melting curve analysis of the PCR products revealed only one melting temperature peak for each amplification reaction, ensuring the specificity of each primer pair. Compared with the protein expression patterns, the mRNA expression of four of the five genes (BB, CC, DD, and EE) exhibited similar patterns, while AA showed different variations (Fig. 10).

## Discussion

Growth of abalone larvae in the trochophore and veliger stages involves many developmental events. However, the underlying molecular mechanisms of development remain largely unknown. The combined use of 2-DE and label-free quantitative proteomics method was adopted. Our combined proteomic approach suggests that novel aspects of the proteins identified are related to shell formation, torsion, nervous system development, and muscle systems.

In this study, only five proteins overlapped between the 2-DE and MS-based label-free results, that may have been caused as follows: Due to the inherent restrictions of 2-DE, the separation of proteins was limited by protein abundance, isoelectric point, molecular weight and hydrophobicity. It was difficult to effectively separate low abundance proteins with high molecular weight (molecular mass > 200 kD), low molecular weight (molecular weight < 8 kD) proteins, very basic proteins, and hydrophobic proteins. In addition, although there are more than 1000 protein spots on the 2-DE, the differential protein remains a concern, we only excised some of gel spots, many gel spots were not excised and identified. So, in this study, the number of identified proteins were limited by 2-DE. On the other hand, the performance of the label-free proteomics method was restricted by limited abalone protein databases. It identified hundreds of proteins but many proteins were not identified. There is relatively limited cDNA information available for abalone and possible improper annotations of abalone genes. Tryptic digestion resulted in acceptable MS/MS spectra with typical peptide signals, but these mass signals did not match any peptides available in the abalone protein databases. In previous studies, there were few overlaps between 2-DE and LC − MS based results [36].

### Proteins associated with Shell formation, torsion, and nervous system development

Shell formation and changes in body shape are important components of abalone development. The 2-DE results showed that at least two proteins (calmodulin (spot 75) [37] and calreticulin (spot 118) [38]) upregulated in the trochophore stage are involved in molluscan shell formation. Calmodulin and calreticulin were also identified in the larvae of the snail *P. canaliculata* during shell development [22] and in the Pacific oyster *C. gigas* [13]. The upregulation of both proteins indicates that they may contribute to the calcification of larval shells. According to the label-free analysis, additional proteins (CaM kinase II alpha, cadherin-like 3 protein, and calponin) involved in calcium ion transport and regulation were upregulated in the veliger stage. These proteins are likely involved in shell formation that occurs between trochophore and veliger larvae. In addition, calreticulin, as a protein-folding-related protein, can bind to misfolded proteins and either correct the protein folding or direct them toward a degradation pathway. Calreticulin constitutes a cellular protein “quality-control” system [39], that is very important for developmental processes.

In the label-free analysis, proteins 28, 84, and 87 were identified as G proteins. G proteins are important for larval metamorphosis of the abalone *Haliotis rufescens* [40]. Protein spots 126 and 150 were identified as 14–3-3 ε by 2-DE gel analysis and 14–3-3 also plays an important role in left–right patterning during amphibian embryogenesis [41]. The 14–3-3 proteins are involved in neurodevelopment and 14–3-3ε and ζ are associated with neurogenesis and neuronal progenitor cell differentiation in the developing brain [42]. Two spots identified as 14–3-3 increased in the veliger stage. Additionally, a real-time PCR assay also revealed upregulation of 14–3-3 ε mRNA expression in veliger larvae (Fig. 10 A growing veliger undergoes a 180° torsion that exerts a considerable effect on the morphology and anatomy of the adult mollusk form. The 14–3-3 may be related to the 180° torsion and nervous system development of abalones.

Calmodulin and the 14–3-3 gamma protein play important roles in the nervous system, these two proteins are activators of tryptophan 5-monooxygenase and tyrosine 3-monooxygenase [13, 43, 44], which are importmant for the biosynthesis of serotonin, noradrenalin and adrenaline, which, as neurotransmitters, are crucial in neuronal activities [13, 32, 33]. The upregulation of the 14–3-3 protein and calmodulin in veligers might suggest their key roles in neuronal function and indicate an enhanced development of the nervous system as *H. diversicolor* transition from the trochophore to the veliger stage. This result is consistent with a study on the early larval development of the Pacific oyster *C. gigas* [13]. The nervous system development and regulation are not yet understood [13]. The identification of 14–3-3 and calmodulin proteins in our study suggests that the activation mechanism of tryptophan 5-monooxygenase and tyrosine 3-monooxygenase in *H. diversicolor* might be similar to that of mammals [13, 43, 44]. In the label-free analysis, protein 6 was identified as a NCAM-related cell adhesion molecule, which was up-regulated 15.7 fold in the veliger stage. The neural cell adhesion molecule (NCAM) plays a pivotal role in neural development, regeneration, synaptic plasticity, and memory processes [45].

In the 2-DE study, protein spots 1 and 3 were identified as ferritin. Ferritin is required for embryonic and larval development [46]. Ferritin is involved in neural development and is required multiple times during development [47]. Ferritin is also involved in shell formation [48]. The results of 2-DE analysis showed a high abundance of ferratin in trochophore larvae (protein spots 1 and 3). Label-free analysis identified protein number 16 as ferritin, which was downregulated in the veliger stage. Our results are consistent across both analyses. Ferritin is likely important for abalone embryo development.

### Proteins associated with muscle systems

Larval retractor muscle appears encapsulated in the trochophore stage and enlarges as the organism enters the veliger stage. This muscle is required to retract the velum and head region into the protective larval shell in the veliger stage. The accessory larval retractor muscle and the velum ring muscle appear during the transition from the trochophore to the veliger stage [49]. Spots 5, and 123 were identified as members of the actin-depolymerizing factor (ADF)/cofilin family by 2-DE analysis. ADF/cofilins depolymerize actin filaments and are essential regulators of actin dynamics. Muscular remodeling is essential for embryo development [50]. The abundance of ADF/cofilin family members was higher in the trochophore stage than in the veliger stage (spots 5 and 123).

In the 2-DE study, protein spot 139 was identified as profilin, which is important for actin filament recycling and cell migration during development [51]. Profilins regulate actin polymerization [52] and can bind to formin proteins, stimulating the rapid addition of actin monomers to the pointed ends of actin filaments [53]. Profilin1 abundance was 15.82 in the trochophore stage and 9.56 in the veliger stage.

In the label-free analysis, protein 20 was identified as calponin, which was up-regulated 11.41 fold in the veliger stage. Calponin 3 participates in actin cytoskeleton-based activities in embryonic development and myogenesis [54].

### Proteins associated with other biological process of embryonic development

Carbohydrates and lipids provide energy and nutrients for embryonic development [55]. We identified several proteins involved in energy metabolism (Additional file 3: Table S3). Glycolysis-related proteins play a key role in the embryonic development of zebrafish and ascidians [21]. In the label-free analysis, most proteins involved in carbohydrate metabolic processes were up-regulated in veliger larvae, including tauropine dehydrogenase, fructose-bisphosphate aldolase, and a mitochondrial malate dehydrogenase precursor. Among the top 31 most abundant proteins identified (Fig. 5c), tauropine dehydrogenase was up-regulated 49.5 fold in the veliger stage. We also noticed a discrepancy between the protein level and the mRNA expression of the fructose-bisphosphate aldolase gene (Fig. 10 Post-translational modification may be responsible for this discrepancy. Changes at the mRNA level may not indicate changes at the protein level [56]. For fructose-bisphosphate aldolase, many modifications, such as phosphorylation [57], glycosylation (in fact, the fructose bisphosphate aldolase has putative glycosylation sites in its peptides) [58], acetylation [59] and protein fragmentation, etc.

The label-free analysis identified three cell death-related proteins (2, 26, 29) involved in cell-cycle regulation, cell communication, or signal transduction. No 29 was identified as poly (ADP-ribose) polymerase (PARP) 4, PARP is a family of proteins involved in cellular processes mainly involving DNA repair and programmed cell death (apoptosis). No 2 was identified as CaM kinase II (CaMKII) alpha. Caspase 9 (a marker of mitochondrial-triggered apoptosis) was significantly reduced in the CaMKII inhibitor (CaMKIIN) transgenic hearts after ischemia reperfusion (I/R) injury [60]. No 26 was identified as prohibitin-2 (PHB2). PHB2, a potential tumor suppressor protein, plays important roles in inhibition of cell cycle progression, transcriptional regulation, apoptosis and the mitochondrial respiratory chain [61]. Morphological changes during development are often dependent on apoptosis [62]. Apoptosis plays a crucial role in organ formation, such as the formation of digits in vertebrates [63]. Cell death is usually accompanied by the elimination of abnormal cells or cells that fail to activate the normal embryonic genome transcriptome [63]. The morphology of the embryo changes significantly from the early trochophore larval stage to the late veliger larval stage, providing support for this idea.

In the label-free analysis, most proteins involved in cell division, cell shape, and differentiation were up-regulated in veliger larvae. Src family kinases have been implicated in many biological processes such as cell adhesion, migration, proliferation and survival. Protein 13 was identified as a Src tyrosine kinase 1, which was up-regulated 19.05 fold in the veliger stage. Src family tyrosine kinases (SFKs) play critical roles in many cell functions by coupling with upstream receptors and cell-adhesion signaling components. SFKs can be activated by integrin and other adhesion receptors, receptor tyrosine kinases, cytokine receptors, G-protein coupled receptors, and immune response receptors [64].

By 2-DE analysis, protein spot 70 was identified as COP9 signalosome subunit 4, isoform 3. It was up-regulated in trochophore larvae (abundance was 3.20 in trochophore larvae and 1.41 in veliger larvae). The COP9 signalosome is a conserved protein complex that functions in the ubiquitin proteasome pathway. Freilich et al. [65] showed that the COP9 signalosome is conserved in invertebrates and has an essential role in animal development. Using label-free analysis, protein number 82 was identified as COP9 signalosome subunit 4, which was also down-regulated in veliger larvae. As the trochophore stage is a key intermediate phase between embryonic and post embryonic development, the COP9 signalosome may be important in ubiquitin-mediated protein degradation.

## Conclusion

This study provides the first comparative analysis of proteomic profiles at different embryonic development stages of *H. diversicolor*. The results suggest that the combined use of 2-DE and label-free quantitative proteomics provides comprehensive proteome profiling, resulting in significant characterization and extensive comparison of the different embryonic development stages of *H. diversicolor*. We documented protein profiles in abalone development related to shell formation and differentiation in the larval retractor muscle, foot mass, and mantle.

## Additional files

Additional file 1: Table S1. Primer sequences for realtime PCR assay. (DOC 31 kb)

Additional file 2: Table S2. Protein identification using MASCOT database searches. (DOC 238 kb)

Additional file 3: Table S3. Differentially abundances 2-DE gel protein spots between trochophore larvae and veliger larvae stage identified by MALDI-TOF–TOF. (DOC 128 kb)

Additional file 4: Table S4. Differentially abundances proteins between trochophore larvae and veliger larvae stage identified by label-free analysis. (DOC 220 kb)

## Abbreviations

2-DE: two dimensional electrophoresis
ACN: acetonitrile
FDR: false discovery rate
GO: Gene Ontology
IEF: isoelectric focusing
IPG: immobilized pH gradient
MALDI: Matrix-assisted laser desorption/ionization
MALDI-TOF / TOF: MALDI / time-of-flight / time-of-flight
MS/ MS: tandem mass spectrometry
S/N: signal-to-noise
TFA: trifluoroacetic acid
