# Supplementary material for: Proteomic analysis of trochophore and veliger larvae development in the small abalone Haliotis diversicolor
Source: BMC Genomics. 2017 Oct 23;18:809. doi: 10.1186/s12864-017-4203-7 (PMC5651566; doi:10.1186/s12864-017-4203-7)
Supplement: Supplementary file 1 — Primer sequences for realtime PCR assay. (DOC 31 kb) [file 12864_2017_4203_MOESM1_ESM.doc]

**Additional file 1: Table S1 Primer sequences for realtime PCR assay**

| Designated name | Primers |
| --- | --- |
| 18sRNA | F TTCCCAGTAAGCGTCAGTCATC  R CGAGGGTCTCACTAAACCATTC |
| Y-box protein 1 | F CCGACTCCGAACAACAACAG  R TTTATGAAGCCATATCCGCTCT |
| fructose-bisphosphate aldolase | F GCTGACCACAATGTCTTCTT  R TCCTGACTGCTGTTCTTCT |
| 14-3-3 ε | F GATGCGATTGCCATGTTAGA  R GTCCACAGCGTAAGGTTATC |
| profilin | F GGAAGAAGCAGCAACGAT  R TCACCATCTGACCTCACAA |
| actin-depolymerizing factor (ADF)/cofilin | F ACAGTGGACAGTGTATCGTA  R CTGGTGAGTTGCGTTGTT |
| calreticulin | F GATGAGATGGATGGAGAATGG  R CCTTTGTAGTCTGGGTTGTC |
