# Supplementary material for: Proteomic analysis of trochophore and veliger larvae development in the small abalone Haliotis diversicolor
Source: BMC Genomics. 2017 Oct 23;18:809. doi: 10.1186/s12864-017-4203-7 (PMC5651566; doi:10.1186/s12864-017-4203-7)
Supplement: Supplementary file 2 — Protein identification using MASCOT database searches. (DOC 238 kb) [file 12864_2017_4203_MOESM2_ESM.doc]

**Additional file 2: Table S2 Protein identification using MASCOT database searches**

| Spots no a) | Protein b) | Species | Accession  No | Protein  MW | Protein  PI | Protein  Score | Protein Score C.I% | Rank Result Type |
| --- | --- | --- | --- | --- | --- | --- | --- | --- |
| 1 | ferritin | *Crassostrea gigas* | gi|40643026 | 20128.6 | 5.05 | 92 | 99.36 | Mascot |
| 2 | Cu，Zn-superoxide dismutase | *Haliotis diversicolor supertexta* | gi|62901684 | 15842.9 | 5.85 | 542 | 100 | Mascot |
| 3 | soma ferritin | *Aplysia californica* | gi|94471616 | 19930.6 | 5.01 | 96 | 99.721 | Mascot |
| 4 | soma ferritin | *Aplysia californica* | gi|94471616 | 19930.6 | 5.01 | 98 | 99.832 | Mascot |
| 5 | actin depolymerisation factor/cofilin | *Haliotis diversicolor* | gi|157072781 | 18306.2 | 6.74 | 219 | 100 | Mascot |
| 6 | ferritin | *Crassostrea gigas* | gi|40643026 | 20128.6 | 5.05 | 98 | 99.843 | Mascot |
| 7 | soma ferritin | *Aplysia californica* | gi|94471616 | 19930.6 | 5.01 | 121 | 100 | Mascot |
| 8 | 60S acidic ribosomal protein P2 | *Haliotis diversicolor* | gi|166406907 | 11419.8 | 4.6 | 301 | 100 | Mascot |
| 9 | soma ferritin | *Aplysia californica* | gi|94471616 | 19930.6 | 5.01 | 102 | 99.931 | Mascot |
| 10 | soma ferritin | *Aplysia californica* | gi|94471616 | 19930.6 | 5.01 | 118 | 99.998 | Mascot |
| 11 | soma ferritin | *Aplysia californica* | gi|94471616 | 19930.6 | 5.01 | 125 | 100 | Mascot |
| 12 | soma ferritin | *Aplysia californica* | gi|94471616 | 19930.6 | 5.01 | 88 | 98.067 | Mascot |
| 13 | soma ferritin | *Aplysia californica* | gi|94471616 | 19930.6 | 5.01 | 128 | 100 | Mascot |
| 14 | ferritin | *Crassostrea gigas* | gi|40643026 | 20128.6 | 5.05 | 150 | 100 | Mascot |
| 15 | ferritin GF1 | *Crassostrea gigas* | gi|32479249 | 20128.7 | 5.15 | 127 | 100 | Mascot |
| 16 | Polyubiquitin | *Elaeagnus umbellata* | gi|3126967 | 51315.6 | 6.38 | 342 | 100 | Mascot |
| 17 | Polyprotein | *Bovine viral diarrhea virus 1-Osloss* | gi|323230 | 449909.1 | 8.83 | 83 | 94.294 | Mascot |
| 18 | peptidyl-prolyl cis-trans isomerase | *Paracoccidioides brasiliensis Pb03* | gi|225677985 | 26498.3 | 8.46 | 95 | 99.679 | Mascot |
| 19 | PREDICTED:similar to putative cytosolic thioredoxinp eroxidase | *Acyrthosiphon pisum* | gi|193615479 | 21723 | 5.67 | 201 | 100 | Mascot |
| 20 | manganese-superoxide dismutase | *Haliotis diversicolor* | gi|166406953 | 18872.4 | 5.95 | 371 | 100 | Mascot |
| 21 | proteasome alpha type 2 | *Haliotis discus discus* | gi|126697376 | 26249.2 | 5.73 | 368 | 100 | Mascot |
| 22 | malate dehydrogenase precursor | *Haliotis discus discus* | gi|126697476 | 26015.8 | 8.91 | 346 | 100 | Mascot |
| 23 | 60S acidic ribosomal protein P0 | *Haliotis diversicolor* | gi|166406846 | 28573.2 | 9.47 | 228 | 100 | Mascot |
| 24 | 60S acidic ribosomal protein P0 | *Haliotis diversicolor* | gi|166406846 | 28573.2 | 9.47 | 796 | 100 | Mascot |
| 25 | glyceraldehyde 3-phosphate dehydrogenase | *Haliotis discus discus* | gi|126697350 | 31627.9 | 6.13 | 194 | 100 | Mascot |
| 26 | fructose 1，6-bisphosphate aldolase | *Haliotis diversicolor* | gi|166406769 | 24196.2 | 7.08 | 275 | 100 | Mascot |
| 27 | hypothetical protein BRAFLDRAFT_260175 | *Branchiostoma floridae* | gi|260798438 | 38518.9 | 5.72 | 183 | 100 | Mascot |
| 28 | phosphatidylinositol transfer protein/retinal degenerationb protein | *Culex quinquefasciatus* | gi|170047812 | 31353.9 | 6.04 | 120 | 99.999 | Mascot |
| 29 | vitellogenin | *Haliotis discus hannai* | gi|164604844 | 265920.8 | 6.66 | 82 | 92.303 | Mascot |
| 30 | PREDICTED:similar to MGC80929 protein isoform 1 | *Strongylocentrotus purpuratus* | gi|72030199 | 34799.8 | 6.32 | 103 | 99.946 | Mascot |
| 31 | esterase | *Idiomarina loihiensis L2TR* | gi|56460874 | 31086.4 | 5.21 | 96 | 99.739 | Mascot |
| 32 | triosephosphate isomerase | *Stylochus sp.KJP-2004* | gi|46909457 | 23371 | 6.33 | 103 | 99.946 | Mascot |
| 33 | prohibitin，putative | *Ixodes scapularis* | gi|241065293 | 29117.2 | 6.02 | 331 | 100 | Mascot |
| 34 | peroxisomal enoyl-coenzyme A hydratase-like protei | *Sus scrofa* | gi|113205878 | 35837.4 | 8.16 | 119 | 99.999 | Mascot |
| 35 | proteasome subunit N3 | *Haliotis discus discus* | gi|126697448 | 28275.1 | 6.84 | 185 | 100 | Mascot |
| 36 | PREDICTED:similar to thiol peroxiredoxin | *Tribolium castaneum* | gi|91092234 | 22026.3 | 5.94 | 94 | 99.536 | Mascot |
| 37 | prevent-host-death family protein | *Deferribacter desulfuricans SSM1* | gi|291278805 | 9566.2 | 8.89 | 83 | 94.915 | Mascot |
| 38 | NADH dehydrogenase subunit I | *Anaplasma marginale str.Puerto Rico* | gi|255003195 | 18402 | 5.96 | 99 | 99.869 | Mascot |
| 39 | Enoyl-CoA hydratase，mitochondrial precursor | *Caligus clemensi* | gi|225717728 | 30116.4 | 8.16 | 148 | 100 | Mascot |
| 40 | triosephosphate isomerase | *Stylochus sp.KJP-2004* | gi|46909457 | 23371 | 6.33 | 97 | 99.793 | Mascot |
| 41 | ubiquitin carboxyl-terminal hydrolase 14 | *Culex quinquefasciatus* | gi|170058708 | 22638.2 | 6.16 | 82 | 92.98 | Mascot |
| 42 | ATP synthase beta subunit | *Haliotis rufenscens* | gi|71370914 | 46238 | 4.9 | 208 | 100 | Mascot |
| 43 | cathepsin L-like cysteine proteinase | *Haliotis diversicolor supertexta* | gi|238481789 | 39765.7 | 5.01 | 91 | 99.116 | Mascot |
| 44 | NADH dehydrogenase iron-sulfur protein 3，mitochondrial precursor | *Rana catesbeiana* | g|226372312 | 29062.1 | 6.62 | 99 | 99.872 | Mascot |
| 45 | charged multivesicular body protein 4c | *Culex quinquefasciatus* | gi|170059579 | 25314.6 | 4.67 | 90 | 98.985 | Mascot |
| 46 | outer membrane protein A precursor | *Shigella flexneri 2a* | gi|38000008 | 35346.6 | 5.31 | 158 | 100 | Mascot |
| 47 | unnamed protein product | *Tetraodon nigroviridis* | gi|47217741 | 37866 | 5.14 | 87 | 97.621 | Mascot |
| 48 | PREDICTED:similar to proteasome-like protein， partial | *Strongylocentrotus purpuratus* | gi|115976544 | 15962 | 5.81 | 95 | 99.623 | Mascot |
| 49 | expressed hypothetical protein | *Trichoplax adhaerens* | gi|196003526 | 36258.8 | 6.55 | 101 | 99.914 | Mascot |
| 50 | receptor of activated kinase C | *Crassostrea angulata* | gi|255918308 | 35158.2 | 7.04 | 144 | 100 | Mascot |
| 51 | fructose 1，6-bisphosphate aldolase | *Haliotis diversicolor* | gi|166406769 | 24196.2 | 7.08 | 193 | 100 | Mascot |
| 52 | glyceraldehyde 3-phosphate dehydrogenase | *Haliotis discus discus* | gi|126697350 | 31627.9 | 6.13 | 379 | 100 | Mascot |
| 53 | Elongation factor Tu，mitochondrial precursor | *Salmo salar* | gi|221221420 | 22912.6 | 7.74 | 95 | 99.656 | Mascot |
| 54 | PREDICTED:hypothetical protein | *Monodelphis domestica* | gi|126273797 | 51265.1 | 8.94 | 182 | 100 | Mascot |
| 55 | glutamate dehydrogenase | *Haliotis discus discus* | gi|126697442 | 28488.6 | 8.94 | 129 | 100 | Mascot |
| 56 | GK21455 | *Drosophila willistoni* | gi|195430526 | 59647.4 | 9.15 | 196 | 100 | Mascot |
| 57 | mitochondrial ATP synthase alpha-subunit | *Cyprinus carpio* | gi|14009437 | 59698.7 | 9.33 | 246 | 100 | Mascot |
| 58 | t complex polypeptide 1 | *Mus musculus* | gi|201725 | 57951.5 | 6.25 | 88 | 98.428 | Mascot |
| 59 | predicted protein | *Nematostella vectensis* | gi|156356135 | 55740.8 | 7.66 | 130 | 100 | Mascot |
| 60 | PREDICTED: similar to SJCHGC09380 protein | *Strongylocentrotus purpuratus* | gi|115696756 | 119751.4 | 6.7 | 252 | 100 | Mascot |
| 61 | inosine 5'-phosphate dehydrogenase 1 | *Mus musculus* | gi|34328209 | 55585.5 | 6.33 | 88 | 98.277 | Mascot |
| 62 | predicted protein | *Nematostella vectensis* | gi|156356135 | 55740.8 | 7.66 | 123 | 100 | Mascot |
| 63 | chaperonin subunit 6a zeta | *Bombyx mori* | gi|114050749 | 58107.7 | 6.59 | 142 | 100 | Mascot |
| 64 | mitochondrial H+ATPase a subunit | *Pinctada fucata* | gi|116008297 | 59813.6 | 8.92 | 661 | 100 | Mascot |
| 65 | GI24428 | *Drosophila mojavensis* | gi|195109418 | 64293.2 | 8.4 | 188 | 100 | Mascot |
| 66 | 60S acidic ribosomal protein P0 | *Haliotis diversicolor]* | gi|166406846 | 28573.2 | 9.47 | 437 | 100 | Mascot |
| 67 | malate dehydrogenase， NAD-dependent[ | *Talaromyces stipitatus ATCC 10500* | gi|242810201 | 35861 | 8.79 | 114 | 99.996 | Mascot |
| 68 | vitellogenin | *Haliotis discus hannai* | gi|164604844 | 265920.8 | 6.66 | 104 | 99.957 | Mascot |
| 69 | PREDICTED:similar to Dnajb11 protein | *Strongylocentrotus purpuratus* | gi|115610690 | 40591 | 5.11 | 180 | 100 | Mascot |
| 70 | PREDICTED:similar to COP9 signalosome subunit 4i soform 3 | *Canis familiaris* | g|74001784 | 43453.2 | 5.58 | 100 | 99.886 | Mascot |
| 71 | HNF-HNF class homeobox protein | *Nematostella vectensis* | gi|93209319 | 98050.2 | 6.02 | 103 | 99.946 | Mascot |
| 72 | similar to pterin-4a-carbinolamine | *Tribolium castaneum* | gi|91087549 | 17246.8 | 9.52 | 142 | 100 | Mascot |
| 73 | PREDICTED:similar to 10 kDa heat shock protein， mitochondrial （Hsp10） （10 kDa chaperonin）（CPN10）isoform 1 | *Macaca mulatta* | gi|109098463 | 11021.9 | 8.89 | 142 | 100 | Mascot |
| 74 | PREDICTED:similar to AGAP007913-PA | *Acyrthosiphon pisum* | gi|193664755 | 9199 | 6.59 | 183 | 100 | Mascot |
| 75 | PREDICTED:similar to calmodulin 2 | *Strongylocentrotus purpuratus* | g i|115728588 | 38469.6 | 4.25 | 142 | 100 | Mascot |
| 76 | PREDICTED:similar to dUTPase | *Ciona intestinalis* | gi|198419421 | 20615.7 | 8.94 | 118 | 99.998 | Mascot |
| 77 | Cu，Zn-superoxide dismutase | *Haliotis diversicolors upertexta* | g i|62901684 | 15842.9 | 5.85 | 491 | 100 | Mascot |
| 78 | Cu，Zn-superoxide dismutase | *Haliotis diversicolors upertexta* | g i|62901684 | 15842.9 | 5.85 | 174 | 100 | Mascot |
| 79 | PREDICTED:similar to dUTPase | *Ciona intestinalis* | gi|198419421 | 20615.7 | 8.94 | 127 | 100 | Mascot |
| 80 | ubiquitin-53aa extension protein | *Spodoptera exigua* | gi|23306794 | 15028 | 9.74 | 87 | 97.88 | Mascot |
| 81 | nucleoside diphosphate kinase B | *Haliotis discus discus* | gi|126697388 | 18912.4 | 8.58 | 255 | 100 | Mascot |
| 82 | translation initiation factor 5A | *Aedes albopictus* | gi|217037905 | 17838.7 | 5.3 | 146 | 100 | Mascot |
| 83 | actin depolymerisation factor/cofilin | *Haliotis diversicolor* | gi|157072781 | 18306.2 | 6.74 | 361 | 100 | Mascot |
| 84 | hypothetical protein | *Trichinella spiralis* | gi|164521906 | 17354.9 | 8.57 | 102 | 99.931 | Mascot |
| 85 | unknown protein 20 | *Haliotis diversicolor* | gi|166406894 | 1916 | 4.94 | 111 | 99.991 | Mascot |
| 86 | PREDICTED:GTP-binding protein SAR1B，putative-like | *Saccoglossus kowalevskii* | gi|291235173 | 21687.1 | 6.21 | 388 | 100 | Mascot |
| 87 | glutathione-S-transferase isoform | *Haliotis diversicolor* | gi|166406777 | 24824.7 | 6.84 | 180 | 100 | Mascot |
| 88 | predicted protein | *Nematostella vectensis* | gi|156405260 | 27611.9 | 6.84 | 96 | 99.714 | Mascot |
| 89 | es1 protein | *Haliotis discus discus* | gi|126697440 | 26938.6 | 6.79 | 157 | 100 | Mascot |
| 90 | ribosomal protein S8 | *Haliotis diversicolor* | gi|166406862 | 24143.1 | 10.53 | 263 | 100 | Mascot |
| 91 | PREDICTED:similar to electron-transfer-flavoprotein，beta polypeptide | *Hydra magnipapillata* | gi|221117102 | 28512.3 | 8.47 | 212 | 100 | Mascot |
| 92 | Enoyl-CoA hydratase，mitochondrial precursor | *Caligus rogercresseyi* | gi|225709666 | 29937.3 | 7.6 | 230 | 100 | Mascot |
| 93 | vacuolar proton-ATPase E-subunit | *Rana catesbeiana* | gi|111924355 | 26063.9 | 7.72 | 141 | 100 | Mascot |
| 94 | l-3-hydroxyacyl-coenzyme a dehydrogenase， short chain | *Haliotis discus discus* | gi|126697366 | 33895.5 | 8.84 | 198 | 100 | Mascot |
| 95 | unnamed protein product | *Mus musculus* | gi|74177575 | 28155.4 | 6.9 | 94 | 99.587 | Mascot |
| 96 | thioredoxin peroxidase 1 | *Haliotis discus discus* | gi|126697354 | 28285.2 | 5.97 | 153 | 100 | Mascot |
| 97 | proteasome Regulatory Particle，Non-ATPase-like family member（rpn-8） | *Caenorhabditis elegans* | g i|17508685 | 40833.1 | 6.03 | 121 | 100 | Mascot |
| 98 | novel protein similar to vertebrate GDP-mannose4，6-dehydratase（GMDS）（zgc:63772） | *Danio rerio* | g i|94734145 | 28725.6 | 8.77 | 123 | 100 | Mascot |
| 99 | hypothetical protein BRAFLDRAFT_98992 | *Branchiostoma floridae* | gi|260828849 | 43913.8 | 5.9 | 284 | 100 | Mascot |
| 100 | isocitrate dehydrogenase 2（NADP+），mitochondrial | *Danio rerio* | gi|41054651 | 50934.7 | 8.35 | 244 | 100 | Mascot |
| 101 | Elongation factor Tu，mitochondrial precursor | *Salmo salar* | gi|221221420 | 22912.6 | 7.74 | 88 | 98.154 | Mascot |
| 102 | Isocitrate dehydrogenase cytoplasmic | *Lepeophtheirus salmonis* | gi|225711968 | 46884.5 | 6.24 | 130 | 100 | Mascot |
| 103 | phosphoglycerate kinase 1 | *Schistosoma japonicum* | gi|226475592 | 26447.8 | 8.85 | 134 | 100 | Mascot |
| 104 | ACYPI004549 | *Acyrthosiphon pisum* | gi|239789408 | 21480.9 | 9.42 | 205 | 100 | Mascot |
| 105 | conserved hypothetical protein | *Candida dubliniensis CD36* | gi|241948403 | 45937.9 | 5.73 | 92 | 99.345 | Mascot |
| 106 | hypothetical protein An08g01960 | *Aspergillus niger* | gi|145239337 | 49548.2 | 5.88 | 81 | 91.94 | Mascot |
| 107 | mitochondrial H+ATPase a subunit | *Pinctada fucata* | gi|116008297 | 59813.6 | 8.92 | 282 | 100 | Mascot |
| 108 | vitellogenin | *Haliotis discus hannai* | gi|164604844 | 265920.8 | 6.66 | 226 | 100 | Mascot |
| 109 | vitellogenin | *Haliotis discus hannai* | gi|164604844 | 265920.8 | 6.66 | 117 | 99.998 | Mascot |
| 110 | vitellogenin | *Haliotis discus hannai* | gi|164604844 | 265920.8 | 6.66 | 109 | 99.986 | Mascot |
| 111 | mitochondrial H+ATPase a subunit | *Pinctada fucata* | gi|116008297 | 59813.6 | 8.92 | 215 | 100 | Mascot |
| 112 | PREDICTED:similar to TATA-binding protein，putative | *Acyrthosiphon pisum* | gi|193713703 | 50570.3 | 6.31 | 225 | 100 | Mascot |
| 113 | PREDICTED:similar to GM12270p | *Nasonia vitripennis* | gi|156546520 | 59672.1 | 5.76 | 154 | 100 | Mascot |
| 114 | predicted protein | *Nematostella vectensis* | gi|156408155 | 55581.5 | 6.9 | 88 | 98.111 | Mascot |
| 115 | methylmalonate semialdehyde dehydrogenase | *Phrynus marginemaculatus* | gi|262305449 | 17261.5 | 6.45 | 114 | 99.996 | Mascot |
| 116 | predicted protein | *Micromonas pusilla CCMP1545* | gi|226455534 | 121392.5 | 5.63 | 82 | 93.886 | Mascot |
| 117 | raminin receptor | *Haliotis discus discus* | gi|126697324 | 35430.6 | 4.84 | 158 | 100 | Mascot |
| 118 | calreticulin | *Crassostrea gigas* | gi|148717307 | 48499.3 | 4.53 | 159 | 100 | Mascot |
| 119 | protein disulfide isomerase | *Haliotis discus discus* | gi|126697420 | 55485.8 | 4.56 | 338 | 100 | Mascot |
| 120 | PREDICTED:GL12416-like | *Oryctolagus cuniculus* | gi|291402475 | 52176.8 | 5.03 | 921 | 100 | Mascot |
| 121 | heat shock cognate protein 70 | *Haliotis diversicolor* | gi|225906407 | 71571.4 | 5.19 | 613 | 100 | Mascot |
| 122 | heat shock protein cognate 3 | *Acyrthosiphon pisum* | gi|242397408 | 72992.6 | 5.19 | 542 | 100 | Mascot |
| 123 | actin depolymerisation factor/cofilin | *Haliotis diversicolor* | gi|157072781 | 18306.2 | 6.74 | 520 | 100 | Mascot |
| 124 | hypothetical protein BRAFLDRAFT_131258 | *Branchiostoma floridae* | gi|260818117 | 17549 | 6.85 | 145 | 100 | Mascot |
| 125 | Enoyl-CoA hydratase，mitochondrial precurso | *Caligus clemensi* | gi|225717728 | 30116.4 | 8.16 | 170 | 100 | Mascot |
| 126 | PREDICTED:3-monooxygenase/tryptophan5-monooxygenase activation protein，gammapolypeptide 2 | *Danio rerio* | gi|125837336 | 28461 | 4.86 | 191 | 100 | Mascot |
| 127 | actin A3 | *Haliotis iris* | gi|60391984 | 41900.9 | 5.29 | 1140 | 100 | Mascot |
| 128 | RecName:Full=Actin | *Lumbricus rubellus* | gi|2829750 | 41581.6 | 5.46 | 327 | 100 | Mascot |
| 129 | PREDICTED:similar to creatine kinase，brain | *Macaca mulatta* | gi|109085011 | 29724 | 7.74 | 133 | 100 | Mascot |
| 130 | arginine kinase | *Fenneropenaeus chinensis* | gi|56182374 | 40389.4 | 5.92 | 138 | 100 | Mascot |
| 131 | PREDICTED:similar to creatine kinase，brain | *Macaca mulatta* | gi|109085011 | 29724 | 7.74 | 147 | 100 | Mascot |
| 132 | Probable citrate synthase 1，mitochondrial precursor | *Lepeophtheirus salmonis* | gi|225713236 | 52614 | 7.25 | 160 | 100 | Mascot |
| 133 | arginine kinase | *Aplysia kurodai* | gi|13647103 | 39645.2 | 6.67 | 112 | 99.993 | Mascot |
| 134 | phosphoglycerate kinase 1 | *Schistosoma japonicum* | gi|226475592 | 26447.8 | 8.85 | 135 | 100 | Mascot |
| 135 | Probable citrate synthase 1，mitochondrial precursor | *Lepeophtheirus salmonis* | gi|225713236 | 52614 | 7.25 | 149 | 100 | Mascot |
| 136 | arginine kinase | *Fenneropenaeus chinensis* | gi|56182374 | 40389.4 | 5.92 | 144 | 100 | Mascot |
| 137 | PREDICTED:similar to gelsolin | *Strongylocentrotus purpuratus* | gi|115918132 | 41049.6 | 5.01 | 93 | 99.442 | Mascot |
| 138 | ATP synthase beta subunit | *Haliotis rufenscens* | gi|71370914 | 46238 | 4.9 | 594 | 100 | Mascot |
| 139 | profilin | *Haliotis diversicolor* | gi|166406771 | 15853.3 | 5.24 | 260 | 100 | Mascot |
| 140 | two-component sensor kinase，probably involved inp hosphate sensing | *candidate division TM7 single-cellisolate TM7c* | gi|167957382 | 68767 | 9.04 | 93 | 99.48 | Mascot |
| 141 | manganese-superoxide dismutase | *Haliotis diversicolor* | gi|166406953 | 18872.4 | 5.95 | 301 | 100 | Mascot |
| 142 | ATP synthase F1，alpha subunit | *Methylobacterium populi BJ001* | gi|188580724 | 55042.8 | 6.31 | 124 | 100 | Mascot |
| 143 | proteasome alpha type 2 | *Haliotis discus discus* | gi|126697376 | 26249.2 | 5.73 | 130 | 100 | Mascot |
| 144 | proteasome subunit N3 | *Haliotis discus discus* | gi|126697448 | 28275.1 | 6.84 | 111 | 99.991 | Mascot |
| 145 | PREDICTED:similar to Dnajb11 protein | *Strongylocentrotus purpuratus* | g i|115610690 | 40591 | 5.11 | 174 | 100 | Mascot |
| 146 | vitellogenin | *Haliotis discus hannai* | gi|164604844 | 265920.8 | 6.66 | 93 | 99.503 | Mascot |
| 147 | PREDICTED:similar to putative mitochondrial ATP synthase alpha subunit | *Acyrthosiphon pisum* | gi|193652614 | 59985.9 | 9.14 | 126 | 100 | Mascot |
| 148 | possible metal-dependent hydrolase | *Gardnerella vaginalis ATCC 14019* | gi|227506951 | 72690.9 | 6.44 | 80 | 89.375 |  |
| 149 | charged multivesicular body protein 4c | *Culex quinquefasciatus* | gi|170059579 | 25314.6 | 4.67 | 106 | 99.973 | Mascot |
| 150 | 14-3-3 epsilon protein | *Bombyx mori* | gi|148298752 | 29766.7 | 4.66 | 149 | 100 | Mascot |

a) Spot number corresponds to the number on the 2DE in Fig. 1C-D.

b) Protein identiﬁed by the de novo sequencing and MASCOT (www.matrixscience.com) from the NCBI nonredundant databa.
