# Supplementary material for: Proteomic analysis of trochophore and veliger larvae development in the small abalone Haliotis diversicolor
Source: BMC Genomics. 2017 Oct 23;18:809. doi: 10.1186/s12864-017-4203-7 (PMC5651566; doi:10.1186/s12864-017-4203-7)
Supplement: Supplementary file 3 — Differentially abundances 2-DE gel protein spots between trochophore larvae and veliger larvae stage identified by MALDI-TOF–TOF. (DOC 128 kb) [file 12864_2017_4203_MOESM3_ESM.doc]

Additional file 4: Table 3 Differentially abundances 2-DE gel protein spots between trochophore larvae and veliger larvae stage identiﬁed by MALDI-TOF–TOF

| **Spots** a) | **protein name** b) | **(Spot volume)%(Mean±SD)** | |  | **expression quantity** c) | **Biological process** | **Cellular component** |
| --- | --- | --- | --- | --- | --- | --- | --- |
|  |  | **the trochophore** | **the veliger stage** | **P-values** |  |  |  |
| 150 | 14-3-3 epsilon protein | 9.43±0.7 | 83.04±4.26 | 8.23E-04 | + | Development | Cytoplasm |
| 126 | PREDICTED:3-monooxygenase/tryptophan5-monooxygenase activation protein,gammapolypeptide 2 | 54.52±0.8 | 87.72±5.84 | 1.04E-02 | + | Development | Mitochondrion |
| 148 | possible metal-dependent hydrolase | 12.16±1.68 | 26.08±2.57 | 1.37E-03 | + | Development | Cytoplasm |
| 56 | GK21455 | 35.16±4.38 | 70.89±5.92 | 1.06E-03 | + | ATP synthesis | Mitochondrion |
| 93 | vacuolar proton-ATPase E-subunit | 11.48±1.73 | 26.71±3.25 | 4.15E-03 | + | ATP synthesis | Mitochondrion |
| 142 | ATP synthase F1,alpha subunit | 4.58±0.69 | 20.27±1.32 | 5.19E-03 | + | ATP synthesis | Mitochondrion |
| 50 | receptor of activated kinase C | 27.06±2.07 | 49.75±3.87 | 3.93E-03 | + | Stress response | Membrane |
| 87 | glutathione-S-transferase isoform | 13.43±2.93 | 29.86±3.99 | 5.00E-02 | + | Stress response | Cytoplasm |
| 141 | manganese-superoxide dismutase | 0.21±0.07 | 6.55±0.65 | 3.00E-03 | + | Stress response | Mitochondrion |
| 60 | PREDICTED: similar to SJCHGC09380 protein | 3.46±0.51 | 7.14±0.72 | 2.50E-02 | + | Energy production and storage | Cytoplasm |
| 135 | Probable citrate synthase 1,mitochondrial precursor | 12.35±1.76 | 24.86±3.91 | 6.09E-02 | + | Energy production and storage | Mitochondrion |
| 140 | two-component sensor kinase,probably involved inp hosphate sensing | 6.91±0.36 | 14.61±2.28 | 3.54E-02 | + | Signal transduction pathway | Membrane |
| 149 | charged multivesicular body protein 4c | 2.95±0.39 | 17.45±1.94 | 3.86E-03 | + | Transport | Endosome |
| 39 | Enoyl-CoA hydratase,mitochondrial precursor | 5.47±0.81 | 21.2±2.78 | 7.23E-03 | + | Electron transport | Mitochondrion |
| 49 | expressed hypothetical protein | 13.85±2.31 | 32.94±4.96 | 6.40E-03 | + | Cell proliferation, development | Cytoplasm |
| 96 | thioredoxin peroxidase 1 | 10.6±1.23 | 6.1±0.64 | 3.99E-02 | - | Stress response | Cytoplasm |
| 36 | PREDICTED:similar to thiol peroxiredoxin | 3.55±0.4 | 0.76±0.13 | 7.07E-03 | - | Stress response | Cytoplasm |
| 2 | Cu,Zn-superoxide dismutase | 47.56±2.53 | 15.43±2.68 | 3.48E-03 | - | Stress response | Cytoplasm |
| 77 | Cu,Zn-superoxide dismutase | 5.49±0.5 | 1.52±0.18 | 3.59E-03 | - | Stress response | Cytoplasm |
| 78 | Cu,Zn-superoxide dismutase | 7.65±0.63 | 0.96±0.13 | 2.06E-03 | - | Stress response | Cytoplasm |
| 84 | hypothetical protein | 6.03±0.29 | 0.14±0.03 | 7.86E-04 | - | Nucleic acid metabolism | Cytoplasm |
| 76 | PREDICTED:similar to dUTPase | 4.87±0.31 | 0.37±0.04 | 1.19E-03 | - | Nucleic acid metabolism | Mitochondrion |
| 79 | PREDICTED:similar to dUTPase | 7.61±0.33 | 0±0 | 6.32E-04 | - | Nucleic acid metabolism | Mitochondrion |
| 61 | inosine 5'-phosphate dehydrogenase 1 | 3.96±0.35 | 0.73±0.06 | 2.90E-03 | - | Nucleic acid metabolism | Cytoplasm |
| 72 | similar to pterin-4a-carbinolamine | 22.27±1.02 | 5.33±0.4 | 7.89E-04 | - | Transcription | Cytoplasm |
| 37 | prevent-host-death family protein | 29.7±1.35 | 17.22±1.65 | 1.39E-03 | - | Transcription | Cytoplasm |
| 1 | Ferritin | 15.58±0.83 | 4.64±0.53 | 1.37E-03 | - | Iron storage | Ferritin complex |
| 3 | soma ferritin | 10.81±0.78 | 2.57±0.21 | 1.59E-03 | - | Iron storage | Ferritin complex |
| 27 | hypothetical protein BRAFLDRAFT_260175 | 22.58±2.41 | 5.63±0.52 | 4.13E-03 | - | Protein degradation | Cytoplasm |
| 41 | ubiquitin carboxyl-terminal hydrolase 14 | 3.51±0.29 | 0.87±0.11 | 3.23E-03 | - | Protein degradation | Proteasome |
| 46 | outer membrane protein A precursor | 20.71±1.18 | 6.96±0.59 | 9.17E-04 | - | Transport | Membrane |
| 45 | charged multivesicular body protein 4c | 43.44±1.4 | 0.45±0.06 | 3.23E-04 | - | Transport | Endosome |
| 118 | Calreticulin | 47.51±1.68 | 3.86±0.28 | 3.45E-04 | - | Calcium ion binding | Endoplasmic reticulum |
| 75 | PREDICTED:similar to calmodulin 2 | 156.2±8.77 | 20.73±3.48 | 5.99E-04 | - | Calcium ion binding | Cytoplasm |
| 5 | actin depolymerisation factor/cofilin | 8.34±0.2 | 0.23±0.03 | 1.66E-04 | - | Cell proliferation,development | Cytoplasm |
| 123 | actin depolymerisation factor/cofilin | 23.11±1.52 | 16.35±1.89 | 3.06E-03 | - | Cell proliferation, development | Cytoplasm |
| 70 | PREDICTED:similar to COP9 signalosome subunit 4i soform 3 | 3.2±0.34 | 1.41±0.14 | 1.48E-02 | - | Development | Signalosome |
| 38 | NADH dehydrogenase subunit I | 7.22±0.89 | 4.26±0.37 | 4.54E-02 | - | Electron transfer | Mitochondrion |
| 69 | PREDICTED:similar to Dnajb11 protein | 19.88±1.55 | 9.89±0.37 | 5.33E-03 | - | Protein folding | Endoplasmic reticulum |
| 40 | triosephosphate isomerase | 6.45±0.58 | 0.79±0.08 | 3.68E-03 | - | Energy production and storage | Cytoplasm |
| 62 | predicted protein | 18.6±0.57 | 6.72±0.6 | 1.02E-04 | - | Molecular chaperone | Membrane |
| 139 | Profilin | 15.82±0.44 | 9.56±0.43 | 1.05E-04 | - | Actin cytoskeleton organization | Cytoskeleton |
| 119 | protein disulfide isomerase | 13.28±0.63 | 4.75±0.41 | 1.69E-03 | - | Cell redox homeostasis | Endoplasmic reticulum |

a) Spot number corresponds to the number on the 2DE in Fig. 1C-D.

b) Protein identiﬁed by the de novo sequencing and MASCOT (www.matrixscience.com) from the NCBI nonredundant databa. ‘+’ showed up-regulated, ‘-‘showed down-regulated.
