# Supplementary material for: Proteomic analysis of trochophore and veliger larvae development in the small abalone Haliotis diversicolor
Source: BMC Genomics. 2017 Oct 23;18:809. doi: 10.1186/s12864-017-4203-7 (PMC5651566; doi:10.1186/s12864-017-4203-7)
Supplement: Supplementary file 4 — Differentially abundances proteins between trochophore larvae and veliger larvae stage identified by label-free analysis. (DOC 220 kb) [file 12864_2017_4203_MOESM4_ESM.doc]

**Additional file 4: Table S4 Differentially abundances proteins between trochophore larvae and veliger larvae stage identiﬁed by label-free analysis**

| NO | **Peak Name** | **Protein name** | **Species** | | **Relative peak intensities^a^**  **C1 C2** | | | **P-values** | **Expression quantity** | **Biological process** | | **Cellular component** |
| --- | --- | --- | --- | --- | --- | --- | --- | --- | --- | --- | --- | --- |
| 47 | cont|000137 | spt|P00760| Cationic trypsin precursor (EC 3.4.21.4) (Beta-trypsin) (Fragment) | *Bos taurus (contaminant)* | | 83.57±5.81 | | 134.65±0.95 | 5.72E-03 | + | Vitelline envelope zona pellucida | | Unclassified |
| 48 | gi|91992392 | vitelline envelope zona pellucida domain 10 | *Haliotis corrugata* | | 308.21±17 | | 783.4±13.48 | 1.05E-04 | + | Vitelline envelope zona pellucida | | Cytoplasm |
| 49 | gi|315441238 | vitelline envelope zona pellucida domain 2 type 1 protein | *Haliotis asinina* | | 76.53±4.94 | | 130.07±7.62 | 8.33E-04 | + | Vitelline envelope zona pellucida | | Cytoplasm |
| 50 | gi|315441240 | vitelline envelope zona pellucida domain 2 type 7 protein | *Haliotis asinina* | | 344.79±21.36 | | 1025.64±29.7 | 3.28E-04 | + | Vitelline envelope zona pellucida | | Cytoplasm |
| 51 | gi|91992368 | vitelline envelope zona pellucida domain 7 | *Haliotis corrugata* | | 26.27±3.09 | | 72.75±4.92 | 1.34E-03 | + | Vitelline envelope zona pellucida | | Cytoplasm |
| 52 | gi|91992376 | vitelline envelope zona pellucida domain 8 | *Haliotis corrugata* | | 5.54±0.44 | | 37.92±4.89 | 6.58E-03 | + | Vitelline envelope zona pellucida | | Cytoplasm |
| 53 | gi|91992378 | vitelline envelope zona pellucida domain 8 | *Haliotis discus hannai* | | 9.18±1.14 | | 60.96±3.95 | 1.22E-03 | + | Vitelline envelope zona pellucida | | Cytoplasm |
| 54 | gi|260408302 | vitelline envelope zona pellucida domain protein 14 | *Haliotis discus hannai* | | 101.42±15.02 | | 562.12±35.14 | 1.48E-03 | + | Vitelline envelope zona pellucida | | Cytoplasm |
| 55 | gi|260408268 | vitelline envelope zona pellucida domain protein 18 | *Haliotis rufescens* | | 12.74±1.85 | | 68.33±5.08 | 1.17E-03 | + | Vitelline envelope zona pellucida | | Cytoplasm |
| 56 | gi|260408274 | vitelline envelope zona pellucida domain protein 21 | *Haliotis rufescens* | | 165.83±6.87 | | 793.92±23.47 | 2.33E-04 | + | Vitelline envelope zona pellucida | | Cytoplasm |
| 57 | gi|260408280 | vitelline envelope zona pellucida domain protein 24 | *Haliotis rufescens* | | 3.01±0.38 | | 15.77±2.11 | 6.85E-03 | + | Vitelline envelope zona pellucida | | Cytoplasm |
| 19 | gi|60391980 | actin A1 | *Haliotis iris* | | 2.46±0.1 | | 4.95±0.44 | 6.56E-03 | + | Muscle contraction and regulation | | Cytoskeleton |
| 20 | gi|156144968 | calponin | *Haliotis diversicolor* | | 0.41±0.07 | | 4.62±0.32 | 1.26E-03 | + | Muscle contraction and regulation | | Cytoskeleton |
| 21 | gi|290751130 | myosin heavy chain type II | *Diodora dysoni* | | 0.91±0.06 | | 3.78±0.33 | 3.52E-03 | + | Muscle contraction and regulation | | Myosin complex |
| 22 | gi|318609972 | paramyosin | *Haliotis discus discus* | | 6.13±0.74 | | 18.13±2.14 | 5.17E-03 | + | Muscle contraction and regulation | | Myosin complex |
| 23 | gi|166406876 | troponin T | *Haliotis diversicolor* | | 8.34±0.59 | | 27.24±3.47 | 7.62E-03 | + | Muscle contraction and regulation | | Troponin complex |
| 39 | gi|73254220 | cytochrome c oxidase subunit 1 | *Reishia clavigera* | | 2.58±0.57 | | 4.1±0.42 | 4.16E-03 | + | Transport | | Mitochondrion |
| 40 | gi|332268269 | ATP synthase F0 subunit 6 | *Haliotis iris* | | 15.38±1.65 | | 39.18±5.83 | 1.19E-02 | + | Transport | | Mitochondrion |
| 41 | gi|29378327 | syntaxin 1A | *Lymnaea stagnalis* | | 3.46±0.35 | | 10.04±2.77 | 4.90E-02 | + | Transport | | Membrane |
| 42 | gi|288938 | RAB2 | *Lymnaea stagnalis* | | 6.06±0.81 | | 20.11±2.2 | 4.73E-03 | + | Transport | | Endoplasmic reticulum |
| 43 | gi|270268071 | selenium-binding protein | *Haliotis discus hannai* | | 0.18±0.01 | | 0.38±0.03 | 3.32E-03 | + | Transport | | Cytoplasm |
| 35 | gi|471279 | KRP-A | *Aplysia californica* | | 84.61±6.49 | | 151.67±8.07 | 2.07E-04 | + | Translation | | Cytoplasm |
| 36 | gi|289919162 | ribosomal protein L10a | *Gibbula varia* | | 0.45±0.05 | | 1.61±0.13 | 2.37E-03 | + | Translation | | Cytoplasm |
| 37 | gi|16755526 | ribosomal protein L26 | *Littorina littorea* | | 0.45±0.04 | | 0.92±0.27 | 7.43E-02 | + | Translation | | Ribosome |
| 38 | gi|126697488 | ribosomal protein l5 | *Haliotis discus discus* | | 258.57±19.61 | | 434.04±29.58 | 1.09E-03 | + | Translation | | Ribosome |
| 12 | gi|410519429 | cathepsin L | *Haliotis discus hannai* | | 1.71±0.11 | | 4.85±0.58 | 1.18E-02 | + | Development | | Lysosome |
| 13 | gi|207339266 | Src tyrosine kinase 1 | *Aplysia californica* | | 9.46±1.14 | | 180.24±15.98 | 2.57E-03 | + | Development | | Membrane |
| 14 | gi|27368649 | H2 | *Haliotis tuberculata* | | 5.89±0.39 | | 14.06±1.61 | 9.46E-03 | + | Development | | Nucleus |
| 8 | gi|126697396 | glutaredoxin 5 | *Haliotis discus discus* | | 4.01±0.71 | | 12.55±2.7 | 1.87E-02 | + | Cell redox homeostasis | | Mitochondrion |
| 9 | gi|326535851 | protein disulfide isomerase | *Conus imperialis* | | 31.26±3.14 | | 442.4±22.91 | 7.71E-04 | + | Cell redox homeostasis | | Endoplasmic reticulum |
| 29 | gi|297186112 | poly [ADP-ribose] polymerase 4 | *Aplysia californica* | | 1.3±0.23 | | 2.07±0.11 | 3.06E-02 | + | Stress response, cell death | | Cytoplasm, Cytoskeleton, Nucleus |
| 30 | gi|111661543 | catalase | *Haliotis diversicolor supertexta* | | 0.99±0.21 | | 12.45±1.74 | 6.90E-03 | + | Stress response | | Periplasm |
| 31 | gi|218683627 | heat shock protein 60 | *Biomphalaria glabrata* | | 156.71±10.43 | | 268.13±19.36 | 5.31E-03 | + | Stress response | | Cytoplasm |
| 3 | gi|20804397 | tauropine dehydrogenase | *Haliotis discus hannai* | | 10.48±1.64 | | 518.76±18.13 | 3.96E-04 | + | Carbohydrate metabolic process | | Cytoplasm |
| 4 | gi|71370900 | fructose-bisphosphate aldolase, partial | *Crepidula fornicata* | | 5.51±0.45 | | 10.18±2.13 | 4.09E-02 | + | Carbohydrate metabolic process | | Cytoplasm |
| 44 | gi|30313537 | mitochondrial malate dehydrogenase precursor | *Littorina littorea* | | 5.15±0.45 | | 37.06±2.66 | 1.59E-03 | + | Tricarboxylic acid cycle | | Mitochondrion |
| 45 | gi|30313533 | mitochondrial malate dehydrogenase precursor | *Nucella freycineti* | | 8±0.87 | | 15.76±0.85 | 5.56E-03 | + | Tricarboxylic acid cycle | | Mitochondrion |
| 5 | gi|71564273 | cadherin like 3 | *Biomphalaria glabrata* | | 5.87±1.02 | | 16.77±1.49 | 7.37E-03 | + | Cell adhesion | | Membrane |
| 6 | gi|13177630 | NCAM-related cell adhesion molecule | *Aplysia californica* | | 0.5±0.11 | | 7.82±1.06 | 5.78E-03 | + | Cell adhesion | | Membrane |
| 27 | gi|4519617 | collagen pro alpha-chain | *Haliotis discus* | | 5.56±0.68 | | 9.19±0.91 | 1.58E-03 | + | Signal transduction | | Collagen trimer |
| 28 | gi|154816325 | small G-protein | *Aplysia californica* | | 1.25±0.2 | | 7.43±0.69 | 3.83E-03 | + | Signal transduction | | Membrane |
| 33 | gi|157930920 | splicing factor arginine/serine-rich 4 | *Haliotis diversicolor supertexta* | | 0.14±0.03 | | 0.28±0.05 | 2.28E-02 | + | Transcription | | Nucleus |
| 34 | gi|65307079 | ELAV 2-like protein | *Aplysia californica* | | 21.46±3.93 | | 49.29±5.66 | 6.53E-03 | + | Transcription, nervous system development | | Cytoplasm |
| 1 | gi|126697378 | mitochondrial ATP synthase delta chain | *Haliotis discus discus* | | 15.66±1.56 | | 29.88±2.25 | 3.24E-03 | + | ATP synthesis coupled proton transport | | Mitochondrion |
| 2 | gi|224458718 | CaM kinase II alpha | *Aplysia californica* | | 14.8±0.81 | | 77.21±4.71 | 1.37E-03 | + | Calcium ion transport,  Neurogenesis, positive regulation of cardiac muscle cell apoptotic process | | Cytoplasm |
| 7 | gi|71679642 | extracellular regulated kinase | *Littorina littorea* | | 1.09±0.21 | | 2.28±0.22 | 7.39E-03 | + | Cell division | | Cytosol |
| 11 | gi|51537343 | cytoplasmic fragile X interacting protein | *Aplysia californica* | | 29.61±3.29 | | 45.41±3.88 | 4.51E-03 | + | Cell shape, Differentiation, Neurogenesis | | Cytoplasm |
| 15 | gi|402170437 | Adh3, partial | *Nucella lapillus* | | 0.69±0.12 | | 2.12±0.28 | 4.28E-03 | + | Ethanol oxidation | | Unclassified |
| 17 | gi|509413 | twitchin-like protein | *Aplysia californica* | | 40.7±3.39 | | 95.95±5.3 | 5.80E-04 | + | Kinase,Transferase | | Cytoskeleton |
| 18 | gi|126697474 | axonemal dynein light chain p33 | *Haliotis discus discus* | | 17.81±2.09 | | 26.78±5.39 | 4.28E-02 | + | Motor protein | | Dynein complex |
| 24 | gi|158997667 | histone 2B | *Aplysia californica* | | 7.01±0.88 | | 12.55±1.39 | 9.69E-03 | + | Nucleosome assembly | | Nucleus |
| 25 | gi|27763677 | eukaryotic translation initiation factor 2 alpha subunit | *Helix aspersa* | | 132.66±9.63 | | 323.7±28.24 | 3.15E-03 | + | Protein biosynthesis | | Cytoplasm |
| 26 | gi|408778253 | prohibitin-2, partial | *Potamopyrgus antipodarum* | | 3.79±0.33 | | 6.19±0.23 | 1.02E-03 | + | Proteolysis,protein folding,replicative cell aging | | Membrane |
| 32 | gi|166079862 | synapse-associated protein | *Aplysia californica* | | 1.31±0.11 | | 2.13±0.29 | 2.52E-02 | + | Synaptic transmission | | Synapse |
| 46 | gi|166406844 | ubiquitin-conjugating enzyme | *Haliotis diversicolor* | | 2.57±0.36 | | 11.67±1.85 | 9.28E-03 | + | Ubl conjugation pathway | | Ubiquitin ligase complex |
| 66 | RRRRRgi|71733128 | REVERSED nonmuscle myosin II, partial | *Aplysia californica* | | 1.98±0.3 | | 1.27±0.17 | 5.78E-02 | - | Muscle contraction and regulation | | Myosin complex |
| 67 | gi|4249742 | myosin II heavy chain | *Ilyanassa obsoleta* | | 21.21±2.93 | | 10.35±0.85 | 1.26E-02 | - | Muscle contraction and regulation | | Myosin complex |
| 68 | gi|29378341 | munc18-1-interacting protein 1 | *Lymnaea stagnalis* | | 1.75±0.16 | | 0.82±0.07 | 3.02E-03 | - | Muscle contraction and regulation | | Unclassified |
| 69 | gi|37544573 | myosin heavy chain | *Littorina littorea* | | 31.62±4.78 | | 14.7±1.68 | 1.36E-02 | - | Muscle contraction and regulation | | Myosin complex |
| 70 | gi|71733128 | nonmuscle myosin II, partial | *Aplysia californica* | | 32.98±3.28 | | 14.32±3.47 | 2.64E-03 | - | Muscle contraction and regulation | | Myosin complex |
| 71 | gi|852074 | myosin regulatory light chain (N-terminus) | *Aplysia californica* | | 11.54±1.73 | | 3.79±0.25 | 1.96E-02 | - | Muscle contraction and regulation | | Cytoplasm, Cytoskeleton |
| 72 | gi|290751152 | myosin heavy chain type II | *Lepetodrilus pustulosus* | | 0.83±0.12 | | 0.1±0.02 | 7.34E-03 | - | Muscle contraction and regulation | | Myosin complex |
| 84 | gi|154816327 | small G-protein | *Aplysia californica* | | 12.15±2.25 | | 6.51±0.13 | 4.44E-02 | - | Signal transduction | | Membrane |
| 85 | gi|269854565 | Cdc24-like protein | *Biomphalaria glabrata* | | 23.73±2.63 | | 3.95±0.16 | 5.25E-03 | - | Signal transduction | | Membrane |
| 86 | gi|68272051 | p38 MAPK | *Biomphalaria glabrata* | | 26.1±2.35 | | 16.81±0.99 | 1.18E-02 | - | Signal transduction | | Cytoplasm,Nucleus |
| 87 | RRRRRgi|109690025 | REVERSED guanine nucleotide-binding protein G(o) alpha subunit | *Aplysia californica* | | 68.02±3.17 | | 21.87±2.68 | 1.10E-04 | - | Signal transduction | | Unclassified |
| 88 | gi|51038265 | thyroid peroxidase-like protein | *Aplysia californica* | | 6.7±0.69 | | 3.77±0.22 | 8.78E-03 | - | Stress response | | Membrane |
| 89 | cont|000121 | alpha-1-antichymotrypsin1 *Sus scrofa* | | | 66.18±5.14 | | 23.13±2.9 | 2.12E-03 | - | Stress response | | Extracellular space |
| 90 | gi|300433302 | arylsulfatase | *Dicathais orbita* | | | 14.68±2.24 | 3.25±0.28 | 1.12E-02 | - | | Stress response | Periplasm |
| 75 | gi|71726735 | histone H4 | *Biomphalaria glabrata* | | | 1259.85±56.43 | 789.26±38.3 | 2.02E-03 | - | | Nucleosome assembly | Nucleus |
| 76 | gi|158997661 | histone macro2A.1 | *Aplysia californica* | | | 81.77±5.01 | 42.46±5.42 | 4.83E-03 | - | | Nucleosome assembly | Nucleus |
| 77 | gi|158997655 | histone 1.1 | *Aplysia californica* | | | 5.2±0.65 | 1.96±0.19 | 9.05E-03 | - | | Nucleosome assembly | Nucleus |
| 93 | gi|215982762 | QM-like protein | *Haliotis diversicolor supertexta* | | | 98.52±7.63 | 65.15±4.96 | 5.92E-03 | - | | Translation | Ribosome |
| 94 | gi|160347070 | ribosomal protein S9 | *Haliotis discus discus* | | | 489.09±18.2 | 307.96±15.65 | 7.06E-05 | - | | Translation | Ribosome |
| 95 | gi|363894957 | putative polyadenylate-binding protein 1, partial | *Haliotis diversicolor* | | | 376.14±23.32 | 179.52±14.12 | 1.78E-03 | - | | Translation | Cytoplasm, Nucleus |
| 96 | gi|409974552 | H(+)-transporting two-sector ATPase alpha subunit, partial | *Theodoxus transversalis* | | | 392.08±20.1 | 251.27±16.45 | 2.28E-04 | - | | Transport | Proton-transporting ATP synthase complex |
| 97 | gi|166406858 | ADP/ATP carrier protein | *Haliotis diversicolor* | | | 293.75±14.79 | 195.19±15.55 | 1.17E-03 | - | | Transport | Mitochondrion |
| 98 | gi|194410718 | hemocyanin | *Haliotis diversicolor supertexta* | | | 154.83±8.92 | 1.99±0.14 | 1.10E-03 | - | | Transport | Extracellular space |
| 101 | gi|5588 | type N4 regulatory subunit of protein kinase A | *Aplysia californica* | | | 19.69±1.96 | 7.96±0.81 | 3.30E-03 | - | | Unclassified | Unclassified |
| 102 | gi|308191606 | Uncharacterized protein 5; Flags: Precursor | | | | 4.64±0.56 | 1.37±0.27 | 2.62E-03 | - | | Unclassified | Unclassified |
| 103 | RRRRRgi|346721863 | REVERSED fibrinogen-related protein 3.2 | *Biomphalaria glabrata* | | | 26.42±4.87 | 6.4±0.52 | 1.73E-02 | - | | Unclassified | Unclassified |
| 58 | gi|356984483 | GST zeta, partial | *Reishia clavigera* | | | 0.45±0.09 | 0.12±0.03 | 1.81E-02 | - | | Amino acid metabolic process | Cytoplasm |
| 59 | gi|30515679 | histidine decarboxylase | *Aplysia californica* | | | 26.42±4.79 | 13.93±1.92 | 2.01E-02 | - | | Amino acid metabolic process | Cytosol |
| 63 | gi|53801569 | NaK-ATPase alpha subunit, partial | *Haliotis tuberculata* | | | 71.4±3.73 | 41.49±3.52 | 7.49E-04 | - | | Hydrolase | Membrane |
| 64 | gi|34484257 | sodium/potassium ATPase alpha subunit | *Onchidella borealis* | | | 12.25±1.47 | 2.36±0.31 | 6.21E-03 | - | | Hydrolase | Membrane |
| 79 | gi|312190468 | elongation factor tu-like protein | *Haliotis discus discus* | | | 0.32±0.06 | 0.21±0.04 | 2.01E-02 | - | | Protein biosynthesis | Unclassified |
| 80 | gi|157072783 | elongation factor 1 alpha | *Haliotis diversicolor* | | | 28.75±2.95 | 15.98±1.74 | 2.98E-03 | - | | Protein biosynthesis | Cytoplasm, Nucleus |
| 60 | gi|7434588 | arginine kinase (EC 2.7.3.3) - Japanese abalone (fragment) | | | | 2.38±0.3 | 0.29±0.04 | 5.07E-03 | - | | Carbohydrate metabolic process | Cytoplasm |
| 61 | gi|126697446 | RAB protein | | *Haliotis discus discus* | | 0.33±0.06 | 0.1±0.02 | 1.37E-02 | - | | Cell cycle, protein transport | Membrane |
| 62 | gi|211998646 | tektin A1 | | *Haliotis asinina* | | 77.28±3.87 | 52.98±4.41 | 6.60E-04 | - | | Cilium biogenesis/degradation | Cilium, Cytoplasm, Cytoskeleton |
| 65 | gi|13647103 | arginine kinase | | *Aplysia kurodai* | | 729.03±30.73 | 411.93±13.23 | 2.18E-03 | - | | Kinase,Transferase | Unclassified |
| 73 | gi|1932827 | pedal peptide precursor protein | | *Helix lucorum* | | 17.7±1.7 | 9.66±0.61 | 6.08E-03 | - | | Neuropeptide | Cytoplasm |
| 74 | gi|125901787 | pol-like protein | | *Biomphalaria glabrata* | | 43.89±4.51 | 9.42±0.63 | 4.44E-03 | - | | Nucleic acid binding | Unclassified |
| 78 | gi|38489224 | NADH dehydrogenase subunit 1 | | *Viviparus georgianus* | | 224.67±22.41 | 8.16±0.53 | 3.54E-03 | - | | Oxidation-reduction process | Mitochondrion |
| 81 | gi|356984346 | proteasome 26S subunit ATPase 4, partial | | *Reishia clavigera* | | 103.24±8.65 | 68.19±3.77 | 6.54E-03 | - | | Protein catabolic process | Proteasome |
| 82 | gi|356984695 | COP9 signalosome subunit 4, partial | | *Reishia clavigera* | | 23.13±4.07 | 3.9±0.21 | 1.47E-02 | - | | Protein deneddylation | Nucleus |
| 83 | gi|166406815 | ribophorin II-like protein | | *Haliotis diversicolor* | | 5.28±0.66 | 2.68±0.24 | 1.24E-02 | - | | Protein N-linked glycosylation | Membrane |
| 91 | gi|356983730 | galectin, partial | | *Reishia clavigera* | | 10.48±1.41 | 4.01±0.19 | 1.49E-02 | - | | Synaptic target recognition | Cytosol |
| 92 | gi|126697436 | transcription factor IIB | | *Haliotis discus discus* | | 28.67±3.97 | 3.06±0.14 | 7.63E-03 | - | | Transcription | Nucleus |
| 99 | gi|6746613 | cytosolic malate dehydrogenase precursor | | *Nucella lapillus* | | 4.76±0.87 | 3.17±0.15 | 6.38E-02 | - | | Tricarboxylic acid cycle | Mitochondrion |
| 100 | gi|157930904 | ubiquitin conjugating enzyme | | *Haliotis diversicolor supertexta* | | 8.84±0.78 | 4.4±0.18 | 7.51E-03 | - | | Ubl conjugation pathway | Cytoplasm,HULC complex,nuclear chromatin |
| 104 | gi|91992324 | vitelline envelope zona pellucida domain 2 | | *Haliotis rufescens* | | 1.78±0.17 | 0.25±0.08 | 4.67E-03 | - | | vitelline envelope zona pellucida | Cytoplasm |
| 10 | gi|326535853 | protein disulfide isomerase | | *Conus betulinus* | | 1.78±0.09 | 0.25±0.08 | 1.08E-03 | - | | Cell redox homeostasis | Endoplasmic reticulum |
| 16 | gi|443298643 | ferritin | | *Concholepas concholepas* | | 5.28±0.35 | 2.68±0.26 | 5.22E-04 | - | | Iron storage | Cytoplasm |

^a^Peptide peak abundance is expressed as the average ratio of intensities of up-regulated or down-regulated proteins at veliger larvae, ‘+’ showed up-regulated, ‘-‘showed down-regulated.
